# Kidney VISTA prevents IFN-**γ**/IL-9 axis–mediated tubulointerstitial fibrosis after acute glomerular injury

**DOI:** 10.1172/JCI151189

**Published:** 2022-01-04

**Authors:** Min-Gang Kim, Donghwan Yun, Chae Lin Kang, Minki Hong, Juhyeon Hwang, Kyung Chul Moon, Chang Wook Jeong, Cheol Kwak, Dong Ki Kim, Kook-Hwan Oh, Kwon Wook Joo, Yon Su Kim, Dong-Sup Lee, Seung Seok Han

**Affiliations:** 1 Department of Biomedical Sciences,; 2 Department of Internal Medicine,; 3 Department of Pathology, and; 4 Department of Urology, Seoul National University College of Medicine, Seoul, Korea.

**Keywords:** Nephrology, Fibrosis, Macrophages, T cells

## Abstract

Severe glomerular injury ultimately leads to tubulointerstitial fibrosis that determines patient outcome, but the immunological molecules connecting these processes remain undetermined. The present study addressed whether V-domain Ig suppressor of T cell activation (VISTA), constitutively expressed in kidney macrophages, plays a protective role in tubulointerstitial fibrotic transformation after acute antibody-mediated glomerulonephritis. After acute glomerular injury using nephrotoxic serum, tubules in the VISTA-deficient (*Vsir–/–*) kidney suffered more damage than those in WT kidneys. When interstitial immune cells were examined, the contact frequency of macrophages with infiltrated T cells increased and the immunometabolic features of T cells changed to showing high oxidative phosphorylation and fatty acid metabolism and overproduction of IFN-**γ**. The *Vsir–/–* parenchymal tissue cells responded to this altered milieu of interstitial immune cells as more IL-9 was produced, which augmented tubulointerstitial fibrosis. Blocking antibodies against IFN-**γ** and IL-9 protected the above pathological process in VISTA-depleted conditions. In human samples with acute glomerular injury (e.g., antineutrophil cytoplasmic autoantibody vasculitis), high VISTA expression in tubulointerstitial immune cells was associated with low tubulointerstitial fibrosis and good prognosis. Therefore, VISTA is a sentinel protein expressed in kidney macrophages that prevents tubulointerstitial fibrosis via the IFN-**γ**/IL-9 axis after acute antibody-mediated glomerular injury.

## Graphical abstract

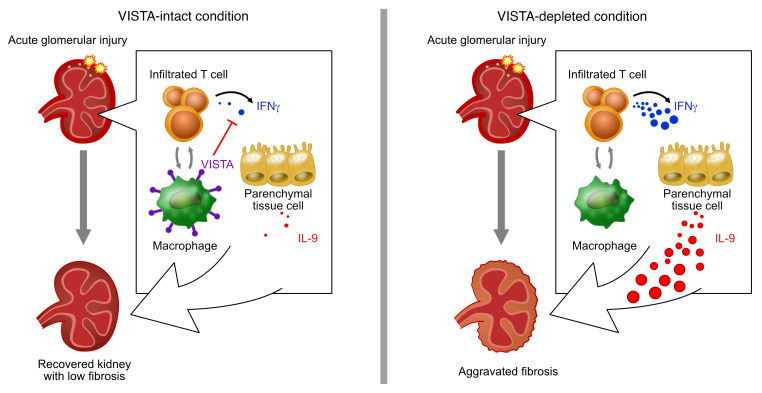

## Introduction

Glomerulonephritis is the most common form of glomerular injury, and if severe, it can lead to tubular dysfunction and leave irreversible fibrotic matrix in the interstitium (1, 2). This pathological process is clinically evident such that heavy proteinuria is a risk factor for chronic kidney disease and end-stage kidney disease (3). Linkage of pathologies between glomerular and tubulointerstitial compartments may be explainable by the fact that filtered proteins in glomeruli have direct toxic effects on tubules if reabsorbed, and these tubules display altered gene signatures such as apoptosis and produce inflammatory cytokines (4). Disrupted glomerulotubular balance and tubuloglomerular feedback can augment the progression of injury, with several mediators, such as reactive oxygen species, chemokines, and cytokines, involved in this process (5, 6). Infiltrated immune cells should play a direct role in the above linkage because these cells are found in the interstitial area surrounding glomeruli and tubules, particularly after severe glomerulonephritis. However, how immune cells participate in this linkage during inflammatory or antiinflammatory processes is not yet fully understood.

There are certain immune cell subsets that reside in nonlymphoid peripheral tissues (named tissue-resident immune cells) and may have a role in the maintenance of immunological homeostasis (7, 8). In particular, tissue-resident macrophages and memory T cells have received attention as the first layer of protection against harmful microorganisms. In mouse kidneys, most resident immune cells are kidney-resident macrophages (9), and their residency in the tubulointerstitial area remains consistent during inflammation (10, 11). Our previous work showed that this macrophage subset had both inflammatory and repair roles after tubular ischemia as a noninfectious injury and functioned by inhibiting overactivation of infiltrated T cells and scavenging dying cells (10). When membrane molecules relevant to this repair function were screened, V-domain Ig suppressor of T cell activation (VISTA) was identified; interestingly, it was highly and constitutively expressed in kidney-resident macrophages and less in kidney-infiltrating macrophages. VISTA is well known to be induced after inflammatory insult or in the tumor environment (12–15), but kidney-resident macrophages substantially express VISTA even under normal conditions without any insult. The present study hypothesized that this frontline molecule may play a protective role in the linkage between glomerular injury and subsequent tubulointerstitial fibrosis. When VISTA was depleted (*Vsir–/–*), tubules suffered more damage in the nephrotoxic nephritis (NTN) model, resulting in acute antibody-mediated glomerular injury. In NTN-induced *Vsir–/–* mice, the contact frequency of macrophages with infiltrated T cells increased and the immunometabolic characteristics of T cells changed, resulting in high oxidative phosphorylation and fatty acid metabolism and overproduction of IFN-γ. Parenchymal IL-9 levels were increased in response to the above immunological actions in the interstitium and generated the fibrotic footprint and consequently kidney dysfunction. Human cases of antineutrophil cytoplasmic autoantibody (ANCA) vasculitis also reflected this pattern such that VISTA expression in biopsied tissues was correlated with low tubulointerstitial fibrosis and improved patient outcomes. Collectively, our results indicate that VISTA, primarily expressed in kidney macrophages, is an inherent molecule that limits tubulointerstitial fibrotic transformation after acute glomerular injury.

## Results

### VISTA expression in the NTN model.

**Figure 1:**
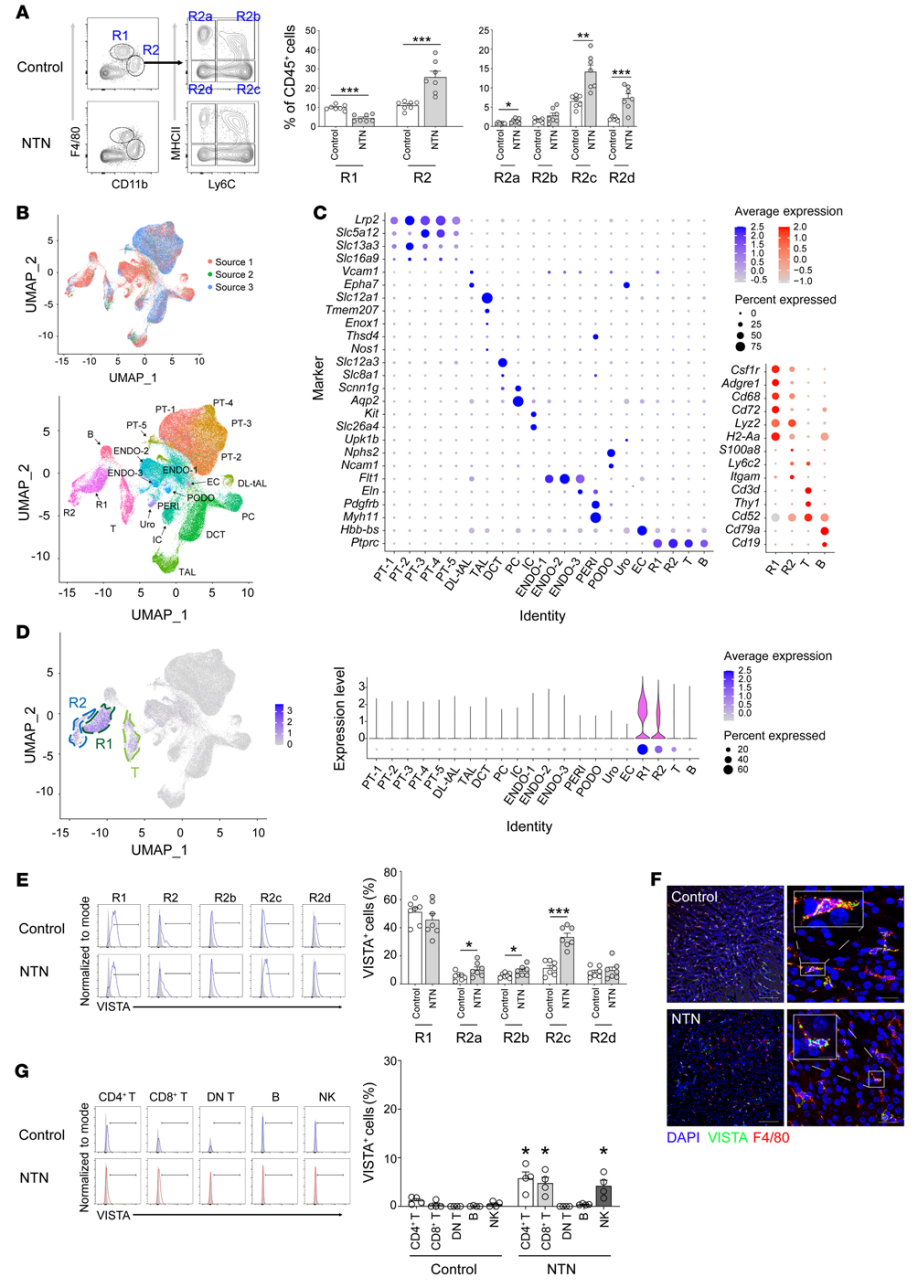
VISTA expression in the NTN model. (**A**) Representative flow cytometry plot of R1 and R2 macrophages. Cells were gated from DAPI^–^CD45^+^Ly6G^–^ subset. Proportions of R1 and R2 macrophages at day 8 after NTN induction (*n* = 7 per group). (**B**) UMAP plots of 86,254 cells pooled from 3 sources with C57BL/6 mice. PT, proximal tubule; DL-tAL, descending limb and thin ascending limb of loop of Henle; TAL, thick ascending limb of loop of Henle; DCT, distal convoluted tubule; PC, principal cell of collecting duct; IC, intercalated cell of collecting duct; ENDO, endothelial cell; PERI, pericyte; PODO, podocyte; Uro, urothelium; EC, erythrocyte. (**C**) Dot plots to identify clusters of parenchymal tissue cells (left) and immune cells (right). (**D**) Violin and dot plots for the expression levels of the *Vsir* gene in clusters. (**E**) Expression of VISTA protein in R1 and R2 macrophages at day 8 after NTN induction (*n* = 7 per group). A representative flow cytometry plot of VISTA is shown. (**F**) Representative image of kidney sections immunostained for DAPI, VISTA, and F4/80. Upper and lower images represent before and after NTN induction, respectively. Scale bar: 100 μm. (**G**) Expression of VISTA protein in other immune cells, such as CD4^+^ T (CD45^+^CD3^+^CD4^+^), CD8^+^ T (CD45^+^CD3^+^CD8^+^), double-negative (DN) T (CD45^+^CD3^+^CD4^–^CD8^–^), B (CD45^+^CD19^+^), and NK (CD45^+^CD3^–^NK1.1^+^) cells (*n* = 4 per group). A representative flow cytometry plot of VISTA after NTN induction is shown. Data are represented as mean ± SEM. *P* values were calculated using an unpaired Student’s *t* test. *P < 0.05; **P < 0.01; ***P < 0.001. Data represent at least 3 independent experiments.

NTN is known to induce immune complex–dependent acute glomerular damage wherein several immune cell subsets, such as macrophages and T cells, participate in this process (16). When the macrophages were categorized into kidney-resident (CD45^+^Ly6G^–^CD11b^int^F4/80^hi^; termed R1) and kidney-infiltrating (CD45^+^Ly6G^–^CD11b^hi^F4/80^int^; termed R2) subsets (10), the proportion of R2 macrophages increased after NTN induction due to infiltration (Figure 1A). Because the surface expression levels of Ly6C and MHC-II are commonly used to characterize subsets of monocytes and macrophages (17, 18), R2 cells were further categorized into 4 subsets by their expression as follows: R2a, Ly6C^–^MHC-II^+^; R2b, Ly6C^+^MHC-II^+^; R2c, Ly6C^+^MHC-II^–^; and R2d, Ly6C^–^MHC-II^–^. Most R2 subsets were highly infiltrated into the inflamed kidneys, except for R2b (Figure 1A).

Within the kidney, VISTA is known to be highly and constitutively expressed in R1 macrophages, but is low and inducible in other immune cell subsets, including R2 macrophages (10). When the transcription level of the *Vsir* gene (gene name of VISTA) was evaluated at the single-cell level (Figure 1, B–D), R1 macrophages had the highest expression, followed by R2 macrophages. Other cells, including T cells and CD45^–^ parenchymal tissue cells, had low and no expression of the *Vsir* gene, respectively.

After inducing NTN injury, approximately 50% of R1 macrophages had consistent expression of VISTA protein (Figure 1E). Regarding R2 macrophages, all subsets had increased expression of VISTA except for the R2d subset. The distribution of macrophages within the kidney is shown in Figure 1F. When other immune cell subsets were evaluated, VISTA expression was increased in CD4^+^ and CD8^+^ T cells and NK cells. However, the expression levels were less than 10% of each subset (Figure 1G). Collectively, VISTA was highly expressed in kidney macrophages (R1 > R2), which remained consistent throughout the NTN model.

### Role of VISTA in the NTN model.

**Figure 2:**
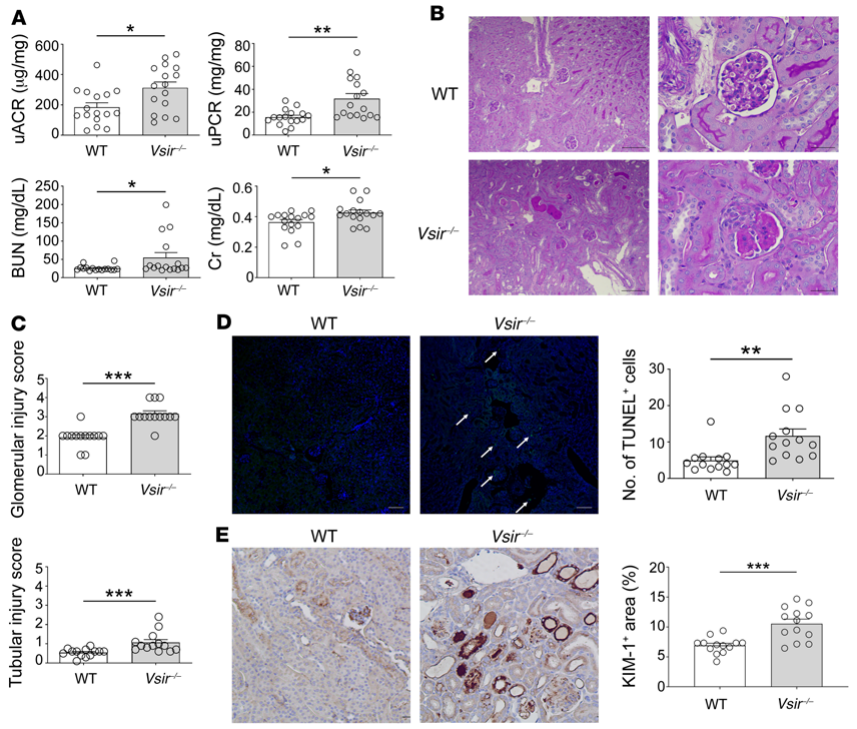
Deleterious parenchymal tissues in VISTA-depleted conditions. (**A**) Kidney damage markers in WT and *Vsir–/–* mice (*n* = 16 per group). uACR, random urine albumin-to-creatinine ratio; uPCR, random urine protein-to-creatinine ratio; BUN, blood urea nitrogen; Cr, creatinine. (**B**) Representative PAS staining images of NTN-induced kidneys. Scale bars: 100 μm (left); 50 μm (right). (**C**) Glomerular and tubular injury scores at day 8 after NTN induction (*n* = 13 per group). (**D**) Representative TUNEL images at day 8 after NTN induction (*n* = 13 per group). TUNEL^+^ cells (arrows) were calculated based on 10 randomly selected images. Original magnification, ×100. (**E**) Representative KIM-1 images at day 8 after NTN induction (*n* = 13 per group). KIM-1^+^ cells were calculated from 10 randomly selected images. Data are represented as mean ± SEM. *P* values were calculated using an unpaired Student’s *t* test. *P < 0.05; **P < 0.01; ***P < 0.001. Data represent at least 3 independent experiments.

To explore the role of VISTA in the NTN model, we compared kidney status between WT and *Vsir–/–* mice. At baseline (before induction), biochemical and histologic differences were not observed between kidneys in WT and *Vsir–/–* mice (Supplemental Figure 1; supplemental material available online with this article; https://doi.org/10.1172/JCI151189DS1). At the early phase after glomerular injury (i.e., day 8), the proteinuria and albuminuria amounts as glomerular injury markers were higher in *Vsir–/–* mice than in WT mice (Figure 2A). Other parameters related to the severity of nephrotic syndrome, such as hypoalbuminemia and hypercholesterolemia, were more severe in *Vsir–/–* mice than in WT mice (Supplemental Figure 2). Markers of tubular injury, such as blood urea nitrogen and serum creatinine, became worse in *Vsir–/–* mice than in WT mice (Figure 2A). When histology was examined, *Vsir–/–* mice had higher scores of glomerular and tubular injury than WT mice, which indicated that glomerular and tubular structural damage, such as crescents, tubular dilatation, and casts, was more frequently observed in *Vsir–/–* mice than in WT mice (Figure 2, B and C). The results of the TUNEL assay and kidney injury molecule-1 positivity supported more damage in *Vsir–/–* mice than in WT mice (Figure 2, D and E).

### Changing characteristics of infiltrating T cells by VISTA depletion.

**Figure 3:**
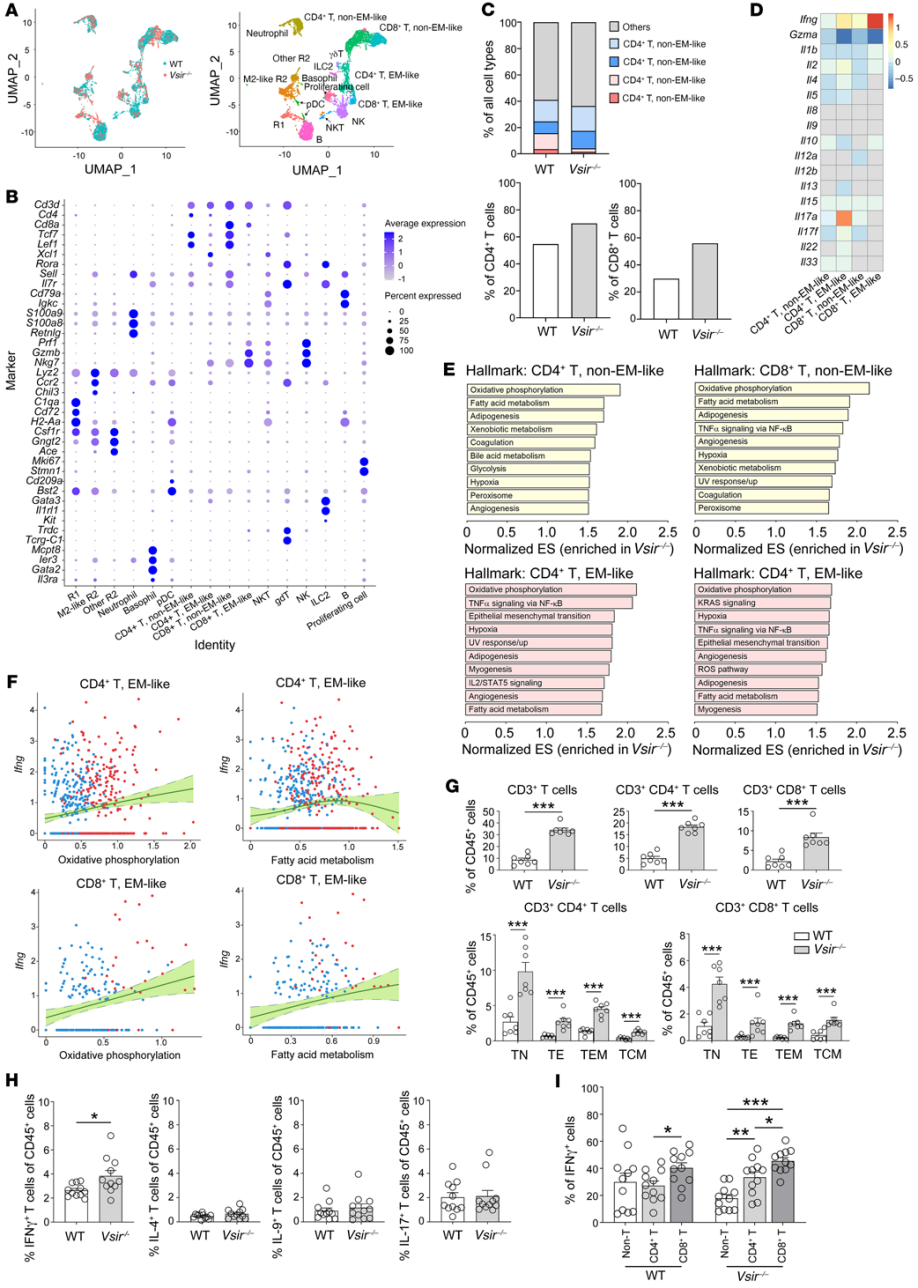
Immunometabolic changes in T cells. (**A**) UMAP plots of 7396 sorted immune cells. (**B**) Dot plot to identify clusters of immune cells. pDC, plasmacytoid dendritic cell; NKT, NK T cell; ILC2, type 2 innate lymphoid cell. (**C**) Proportions of all cell types in WT and *Vsir–/–* kidneys (upper). Proportions of EM-like T cells within each T cell lineage (lower). (**D**) Heatmap of logarithmic fold changes of cytokine genes in T cells from *Vsir–/–* mice compared with WT mice. (**E**) Hallmark gene set enrichment analysis in order of normalized enrichment score (ES). (**F**) Correlation between metabolic features and *Ifng* level in EM-like T cells. Line and area represent nonlinear relationship and 95% CIs, respectively. Blue and red dots represent T cells obtained from the kidneys of WT and *Vsir–/–* mice, respectively. (**G**) Proportions of T cell subsets evaluated by flow cytometry (*n* = 7 per group). TN, naive T cells; TEM, effector memory T cells; TE, effector T cells; TCM, central memory T cells. (**H**) Proportions of cytokine-producing T cells evaluated by flow cytometry (*n* = 11 per group). (**I**) Proportions of IFN-γ^+^ cells in CD4^+^, CD8^+^, and other immune cells (*n* = 11 per group). *P* values were calculated using an unpaired Student’s *t* test for 2 groups or ANOVA with Tukey’s test for multiple comparison. *P < 0.05; **P < 0.01; ***P < 0.001. Data represent 2 or 3 independent experiments.

VISTA on myeloid cells confers negative signals to the T cells in a contact manner (12–15) because T cell proliferation and activation were reduced in VISTA-coated in vitro conditions compared with control-coated conditions (10, 13, 19). To evaluate the T cell status under in vivo NTN conditions, gene profiling was compared between WT and *Vsir–/–* mice using single-cell RNA (scRNA) sequencing. Among immune cells, CD4^+^ and CD8^+^ T cells were clustered into non–effector memory–like (non–EM-like) and EM-like T cells (Figure 3, A and B). The proportions of all clusters are shown in Figure 3C. The relative proportion of the EM-like subset per T cell lineage was higher in *Vsir–/–* mice than in WT mice (Figure 3C). EM-like CD4^+^ T cells of *Vsir–/–* mice expressed *Ifng* and *Il17a* at higher levels than those of WT mice, whereas EM-like CD8^+^ T cells of *Vsir–/–* mice expressed *Ifng* at higher levels than those of WT mice (Figure 3D). The common changes in gene sets of T cell clusters in *Vsir–/–* mice (vs. WT mice) were oxidative phosphorylation and fatty acid metabolism (Figure 3E and Supplemental Figure 3). The tendencies of these metabolic features were positively correlated with *Ifng* levels in EM-like T cells (Figure 3F). Because metabolic signatures are associated with specific immune cell fates and functions (20, 21), metabolic deviation by depletion of VISTA might induce different cytokine profiles in T cells.

When these results were validated by flow cytometry, more CD4^+^ and CD8^+^ T subsets infiltrated the kidneys of *Vsir–/–* mice than those of WT mice (Figure 3G). When their production of cytokines was evaluated using flow cytometry, IFN-γ was primarily different between WT and *Vsir–/–* mice, and its production was most attributable to CD8^+^ T cells (Figure 3, H and I). The use of VISTA-blocking antibody increased IFN-γ production from infiltrated T cells (Supplemental Figure 4), whereas the treatment with inhibitors of oxidative phosphorylation and fatty acid metabolism decreased the production (Supplemental Figure 5), all of which supports the previous results. Collectively, cytokine profiling of T cells was altered in *Vsir–/–* mice after NTN induction, and the most primary feature was high IFN-γ production by metabolically reprogrammed, EM-like CD8^+^ T cells.

### Interaction between macrophages and T cells.

**Figure 4:**
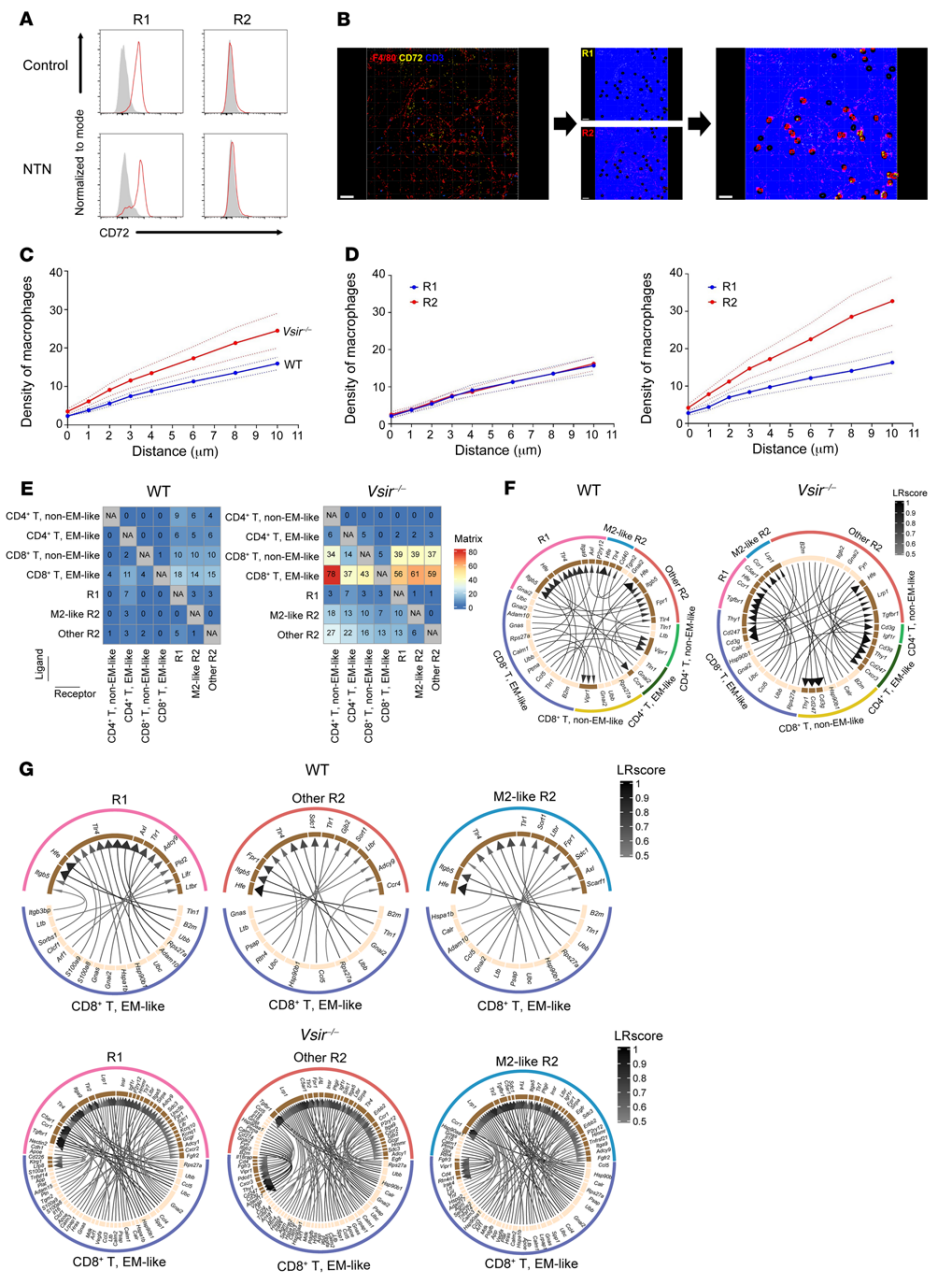
Contact between macrophages and T cells. (**A**) Representative flow cytometry plot of CD72 in R1 and R2 macrophages. (**B**) Representative plot of kidney sections immunostained for F4/80, CD72, and CD3 and analysis process to identify the contact frequency between macrophages and T cells. Scale bars: 50 μm. (**C**) Density of macrophages around T cells. (**D**) Density of macrophage subsets in WT (left) and *Vsir–/–* (right) mice. (**E**) Numbers of ligand-receptor pairs between macrophage and T cell clusters. (**F**) Chord diagrams of the top 30 interactions between macrophage and T cell clusters. The ligand-receptor interaction scores (LR scores) were bounded by 0 and 1, and a high score represented a strong interaction. (**G**) Chord diagrams of all ligand-receptor pairs between EM-like CD8^+^ T cells and each macrophage cluster (see the raw data in Supplemental Data 1). Data represent 2 or 3 independent experiments.

The NTN model is known to be built by the stepwise effects of initial macrophages and subsequent infiltrated T cells (16). The present injury was also dependent on the T cell effect because kidney function was preserved in T cell–depleted mice compared with nondepleted mice (Supplemental Figure 6). Herein, we hypothesized that the contact frequencies between macrophages and T cells differed between WT and *Vsir–/–* mice. R1 and R2 macrophages were detected as F4/80^+^CD72^+^ and F4/80^+^CD72^–^ cells, respectively, whereas T cells were defined as CD3^+^ cells in confocal images (Figure 4, A and B). When the contact frequency was evaluated, *Vsir–/–* mice had a higher density of macrophages around T cells than WT mice (Figure 4C). The high contact frequency in *Vsir–/–* mice was primarily attributable to infiltrating R2 macrophages compared with resident R1 macrophages (Figure 4D). In addition to the contact frequency, intercellular communication between macrophages and T cells was evaluated using ligand-receptor pairs from scRNA-sequencing data. The pair number among all macrophage and T cell clusters was 693 in *Vsir–/–* mice, which was higher than the pair number of 163 in WT mice (Figure 4E). This difference was most prominent at the ligand end of EM-like CD8^+^ T cells. Within the top 30 strong pairs, interactions between R1 macrophages and T cells were more identified in WT mice, whereas those between R2 macrophages and T cells were more identified in *Vsir–/–* mice (Figure 4F). When the interaction with EM-like CD8^+^ T cells was evaluated alone, the strongest pairs were *B2m* (EM-like CD8^+^ T) and *Hfe* (other R2) in WT mice and *B2m* (other R2) and *Cd3g* (EM-like CD4^+^ T) in *Vsir–/–* mice (Figure 4G and Supplemental Data 1). Collectively, the changes in contact frequency and intercellular communication in *Vsir–/–* mice might affect the immunometabolic features of EM-like CD8^+^ T cells.

### Fibrotic response of kidney tissue cells by VISTA depletion.

**Figure 5:**
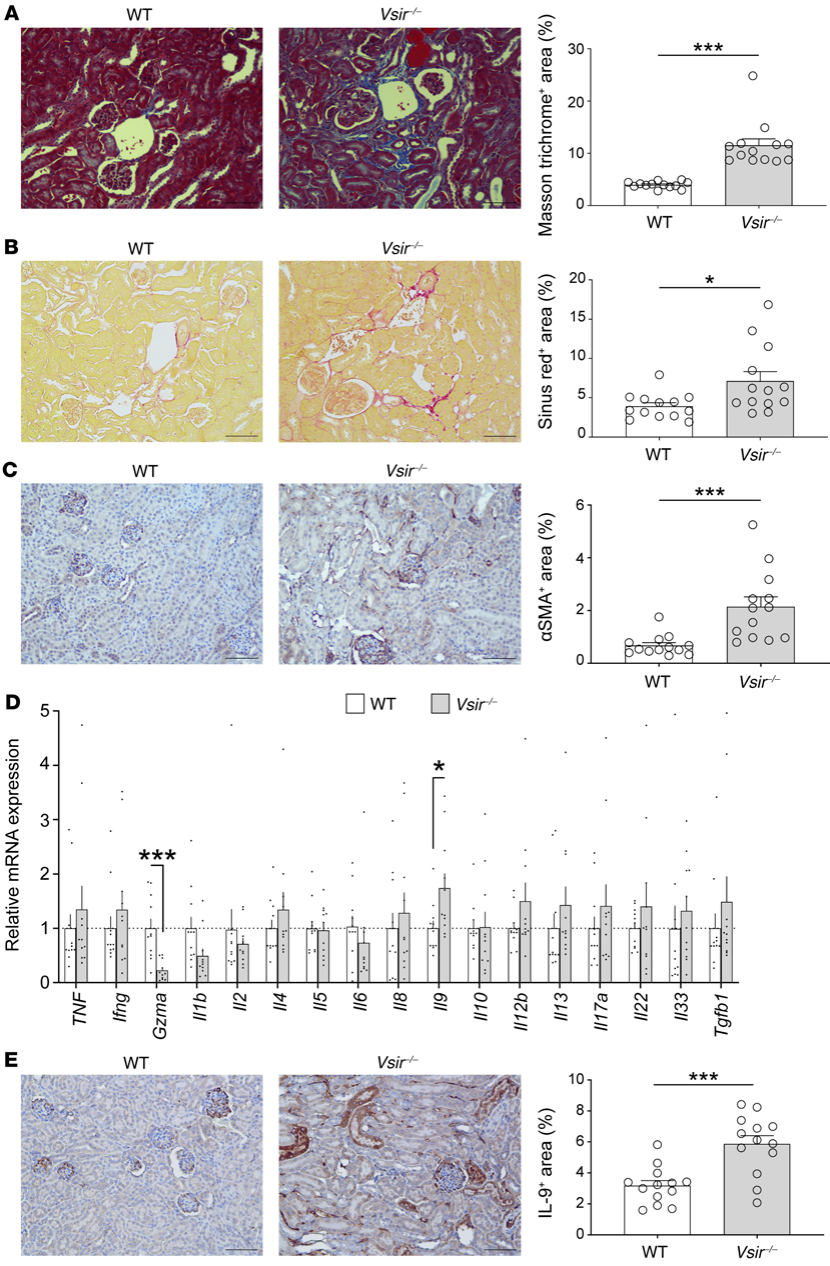
Fibrotic parenchyma in VISTA-depleted kidneys. (**A**) Masson trichrome–positive area in WT and *Vsir–/–* kidneys and their representative plots. Scale bars: 100 μm. (**B**) Sirius red–positive area WT and *Vsir–/–* kidneys and their representative plots. Scale bars: 100 μm. (**C**) αSMA-positive area in WT and *Vsir–/–* kidneys, and their representative plots. Scale bars: 100 μm. (**D**) Cytokine-gene expression of kidney parenchyma. (**E**) Representative plots of kidney sections immunostained for IL-9 and comparison of the IL-9^+^ area between WT and *Vsir–/–* kidneys. Scale bars: 100 μm. Data are represented as mean ± SEM (*n* = 11–13 per group). *P* values were calculated using an unpaired Student’s *t* test. *P < 0.05; ***P < 0.001. Data represent at least 3 independent experiments.

The common final pathology after kidney inflammation is tubulointerstitial fibrosis (22, 23). A consensus exists that infiltrated R2 macrophages play a role in inflammation-induced fibrosis (24, 25), and subsequent activation of infiltrated T cells mediates its progression (26, 27). Nevertheless, it is not completely understood which molecules are inherently protective or mediated in the pathologic process between glomerular injury and tubulointerstitial fibrosis. *Vsir–/–* mice had a higher fibrotic area in the tubulointerstitium than WT mice (Figure 5, A and B). α-Smooth muscle actin, a marker of myofibroblasts, was also more highly expressed in the tubulointerstitium of *Vsir–/–* mice than in that of WT mice (Figure 5C). To evaluate fibrosis-mediating factors in response to immune cell activity, the gene expression levels of several cytokines were compared between parenchymal tissue cells of WT and *Vsir–/–* kidneys (Figure 5D). Among them, *Il9* was more highly transcribed in the parenchymal cells of *Vsir–/–* mice, whereas *Gzma* was less transcribed in *Vsir–/–* parenchymal cells than in WT cells. Levels of expression of other genes, such as *Il4*, *Il12*, *Il13*, and *Tgfb1*, seemed to be higher in *Vsir–/–* mice than in WT mice, but statistical significance was not shown. The protein level of IL-9, which was primarily expressed in glomeruli, interstitium, and some tubules, was higher in the *Vsir–/–* kidney than in the WT kidney (Figure 5E). The protein expression of the receptor for IL-9 did not differ between WT and *Vsir–/–* kidneys (Supplemental Figure 7).

**Figure 6:**
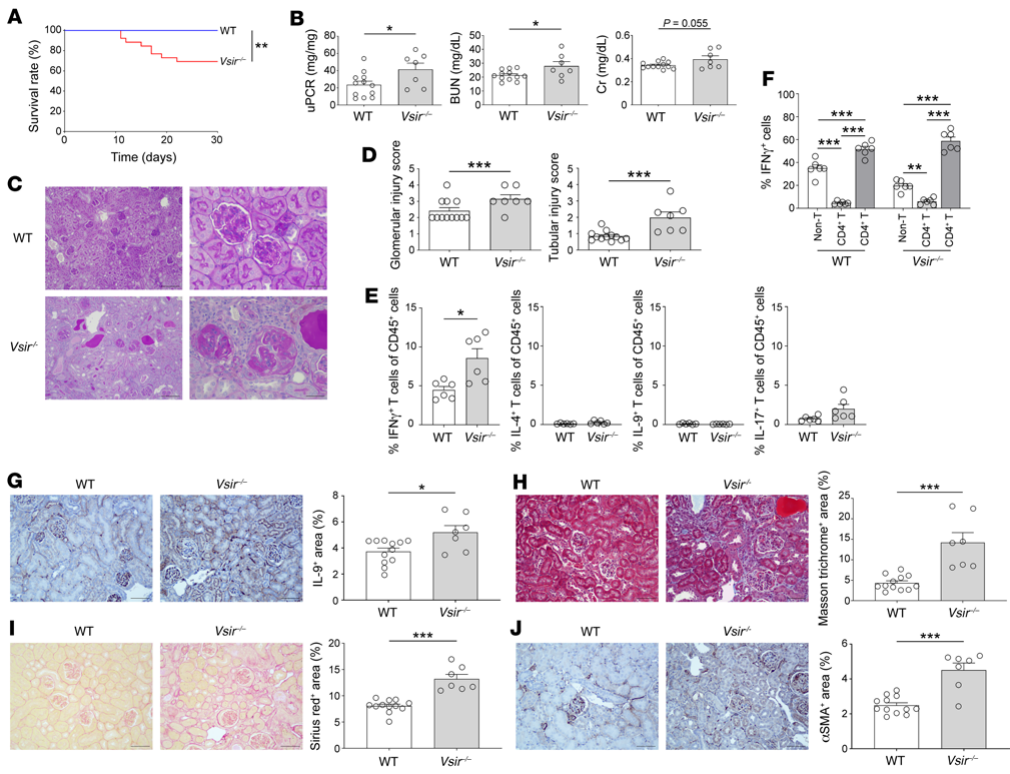
Overwhelming of the IFN-γ/IL-9 axis in the late phase of VISTA-depleted kidneys. (**A**) Survival rates of NTN-induced WT and *Vsir–/–* mice (*n* = 12 per group). Statistical significance for survival was calculated using the Kaplan-Meier method with the log-rank test. (**B**) Kidney damage markers in WT and *Vsir–/–* mice that had survived to day 36 after NTN induction. (**C**) Representative PAS-staining images of kidneys at day 36 after NTN induction. Scale bars: 100 μm (left); 50 μm (right). (**D**) Glomerular and tubular injury scores at day 36 after NTN induction. (**E**) Proportions of cytokine-producing T cells evaluated by flow cytometry. (**F**) Proportions of IFN-γ^+^ cells in CD4^+^, CD8^+^, and other immune cells. (**G**) Representative plots of kidney sections immunostained for IL-9 and comparison of the IL-9^+^ area between WT and *Vsir–/–* kidneys. (**H**) Representative Masson trichrome plots of kidney sections and comparison between WT and *Vsir–/–* kidneys. (**I**) Representative Sirius red plots of kidney sections and comparison between WT and *Vsir–/–* kidneys. (**J**) Representative Sirius red plots of kidney sections immunostained for αSMA, and comparison between WT and *Vsir–/–* kidneys. Data are represented as mean ± SEM (*n* = 7–12 per group). Scale bars: 100 μm. *P* values were calculated using an unpaired Student’s *t* test for 2 groups or ANOVA with Tukey’s test for multiple comparison. *P < 0.05; **P < 0.01; ***P < 0.001. Data represent 2 or 3 independent experiments.

When the NTN-induced mice were followed up until the late phase (>1 month), the death rate was higher in the *Vsir–/–* mice than in the WT mice (Figure 6A). The biochemical and histological damage markers were worse in the *Vsir–/–* mice than in the WT mice (Figure 6, B–D). IFN-γ–producing CD8^+^ T cells were more highly identified in the *Vsir–/–* mice than in the WT mice (Figure 6, E and F). The IL-9^+^ and fibrotic areas were also larger in the *Vsir–/–* mice than in the WT mice (Figure 6, G–J). These results indicate that the IFN-γ/IL-9 axis was overwhelmed even in the late phase of *Vsir–/–* mice compared with WT mice, which determined their tubulointerstitial fibrosis and kidney dysfunction.

### Response of kidney tissues to IFN-γ and IL-9.

**Figure 7:**
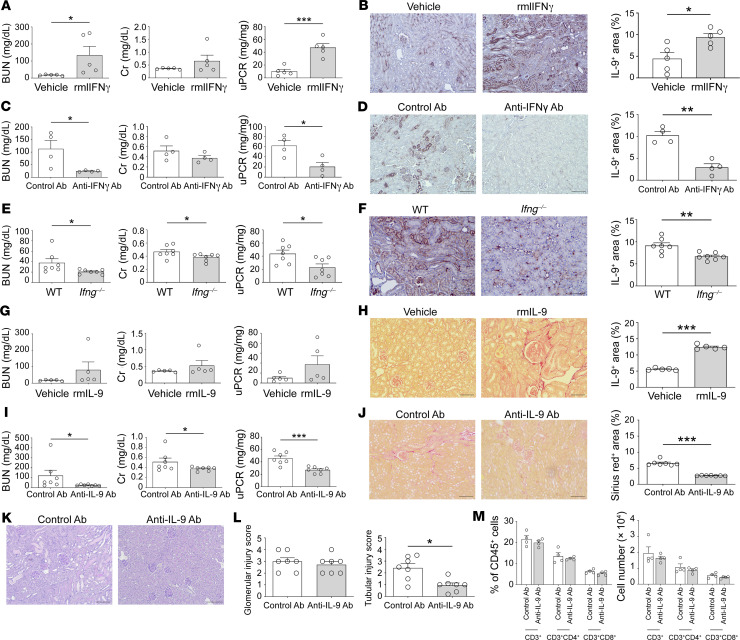
Confirmation of the IFN-γ/IL-9 fibrosis axis. (**A**) Kidney damage markers in vehicle- and rmIFN-γ-treated WT kidneys. (**B**) Representative plots of kidney sections immunostained for IL-9 and comparison of the IL-9^+^ area between vehicle- and rmIFN-γ–treated kidneys. (**C**) Kidney damage markers in control antibody- and anti–IFN-γ antibody–treated *Vsir–/–* kidneys. (**D**) Representative plots of kidney sections immunostained for IL-9 and comparison of the IL-9^+^ area between control antibody- and anti–IFN-γ antibody–treated kidneys. (**E**) Kidney damage markers in NTN-induced *Rag1–/–* mice with adoptive transfer of naive CD8^+^ T cells from WT and *Ifng–/–* mice. (**F**) Representative plots of kidney sections immunostained for IL-9 and comparison of the IL-9^+^ area in NTN-induced *Rag1–/–* kidneys between WT mouse-derived and *Ifng–/–* mouse–derived naive CD8^+^ T cell transfer. (**G**) Kidney damage markers in vehicle- and rmIL-9–treated WT kidneys. (**H**) Representative plots of kidney sections immunostained for Sirius red and comparison of the Sirius red^+^ area between vehicle- and rmIL-9-treated WT kidneys. (**I**) Kidney damage markers in control antibody- and anti–IL-9 antibody–treated *Vsir–/–* kidneys. (**J**) Representative plots of kidney sections immunostained for Sirius red and comparison of the Sirius red^+^ area between control antibody– and anti–IL-9 antibody–treated *Vsir–/–* kidneys. Scale bars: 100 μm. (**K**) Representative PAS staining images of control antibody- and anti–IL-9 antibody–treated *Vsir–/–* kidneys. Scale bars: 100 μm. (**L**) Glomerular and tubular injury scores in control antibody– and anti–IL-9 antibody–treated *Vsir–/–* kidneys. (**M**) Proportions of T cell subsets in control antibody– and anti–IL-9 antibody–treated *Vsir–/–* kidneys. Data are represented as mean ± SEM (*n* = 4–7 per group). *P* values were calculated using an unpaired Student’s *t* test. *P < 0.05; **P < 0.01; ***P < 0.001. Data represent 2 or 3 independent experiments.

To confirm the IFN-γ/IL-9 axis observed in *Vsir–/–* mice, recombinant mouse IFN-γ (rmIFN-γ) and anti–IFN-γ antibodies were administered via subcapsular and intravenous injection, respectively, to NTN-induced mice. The rmIFN-γ–treated mice had higher kidney damage markers than vehicle-treated mice (Figure 7A). rmIFN-γ treatment increased the IL-9^+^ area of the kidneys (Figure 7B). The anti–IFN-γ antibody abrogated these pathologic changes (Figure 7, C and D). We further carried out adoptive transfer of T cells (from WT mice vs. *Ifng–/–* mice) to the NTN-induced *Rag1–/–* mice, and their biochemical damage markers and IL-9 expression were compared (Figure 7, E and F). IFN-γ–producing T cells resulted in more damage and IL-9 production from the parenchyma than nonproducing T cells.

To confirm the IL-9/fibrosis axis observed in *Vsir–/–* mice, recombinant mouse IL-9 (rmIL-9) and anti–IL-9 antibodies were administered via subcapsular and intravenous injection, respectively, to NTN-induced mice. The rmIL-9–treated mice had more fibrotic kidneys than vehicle-treated mice, although the differences in damage markers such as blood urea nitrogen, creatinine, and proteinuria were not prominent between the 2 groups (Figure 7, G and H). When an anti–IL-9 antibody was used, all markers of damage and fibrosis were decreased (Figure 7, I and J). The reduction in histological injury scores by anti–IL-9 antibody was prominent in the tubulointerstitial compartment rather than in the glomerular compartment (Figure 7, K and L). The number and proportion of infiltrated T cells were not different between mice administered control and anti–IL-9 antibodies (Figure 7M). These results suggest that IL-9 affected tubulointerstitial fibrosis without changing the glomerular structure or T cell infiltration. Collectively, the IFN-γ/IL-9 axis participated in the progressive fibrosis of the tubulointerstitium after NTN induction, and VISTA may play a preventive role in this process as a sentinel molecule.

### Impact of VISTA-depleted T cells in the model.

**Figure 8:**
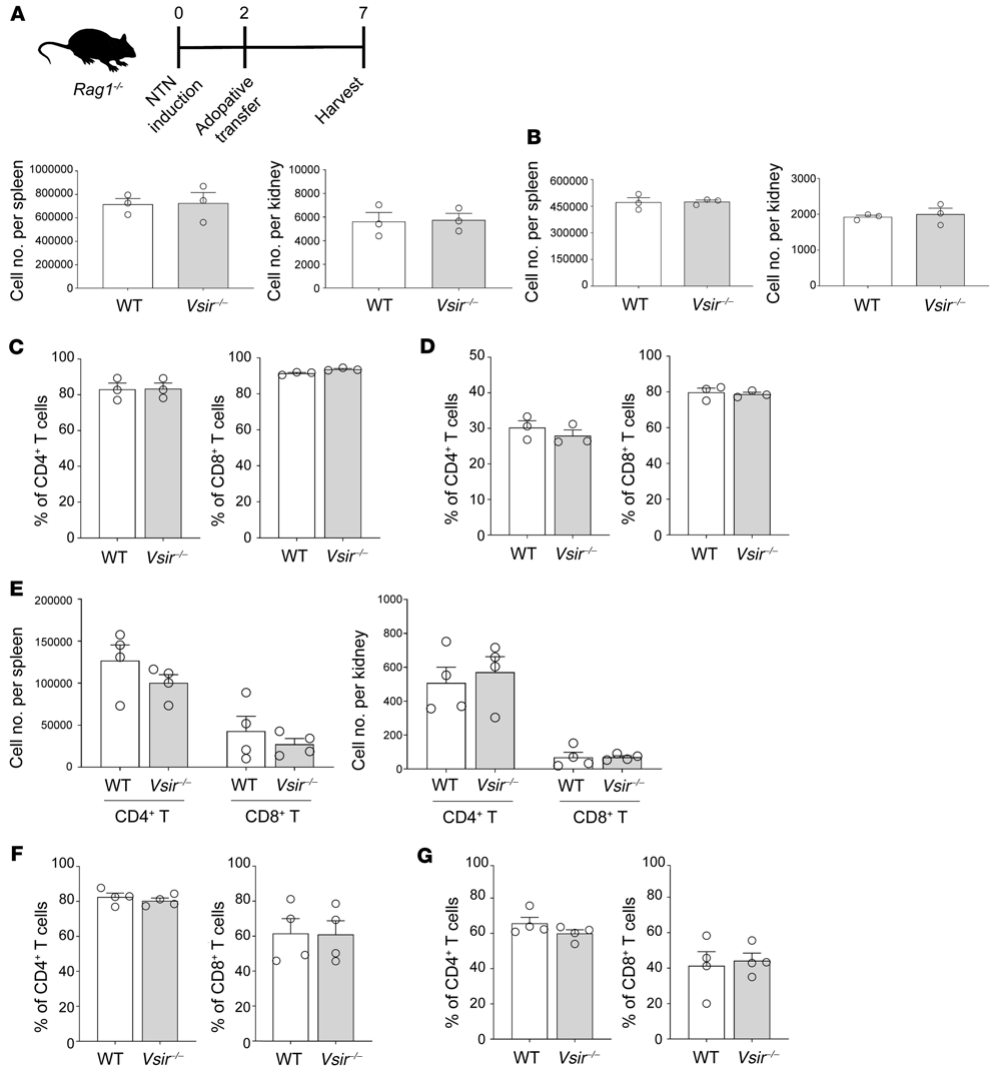
Minimal effects of VISTA-depleted T cells in the NTN model. (**A**) Schematic of adoptive transfer experiments. Infiltrated cell numbers in the spleen and kidney after adoptive transfer of naive CD4^+^ T cells to NTN-induced *Rag1–/–* mice. (**B**) Infiltrated cell numbers in the spleen and kidney after adoptive transfer of naive CD8^+^ T cells to NTN-induced *Rag1–/–* mice. The adoptive transfer scheme was the same as in **A**. (**C**) Proportion of CD44^+^ cells among each T cell lineage in the spleen. (**D**) Proportion of CD44^+^ cells among each T cell lineage in the kidney. (**E**) Infiltrated cell numbers of WT mouse– and *Vsir–/–* mouse–derived CD3^+^ T cells after NTN induction in a competitive assay. The adoptive transfer scheme was the same as in **A**. (**F**) Proportion of CD44^+^ cells among each T cell lineage in the spleen. (**G**) Proportion of CD44^+^ cells among each T cell lineage in the kidney. Data are represented as mean ± SEM (*n* = 3–4 per group). *P* values were calculated using an unpaired Student’s *t* test. All the differences between groups were not significant.

VISTA is also expressed in naive T cells to maintain their quiescence and tolerance, which has been evaluated in lipopolysaccharide injection and graft-versus-host disease models (28). Because the protein expression of VISTA in mouse kidneys was expressed mostly in kidney macrophages (R1 > R2), the NTN results might be primarily attributable to the effects from the macrophage end rather than the T cell end. To determine the VISTA effect at the end of the T cell subset alone, NTN was induced in *Rag1–/–* mice, and the activity of adoptively transferred *Vsir–/–* T cells was evaluated in comparison with that of WT T cells. Referentially, adoptive transfer of T cells to NTN-induced *Rag1–/–* mice was sufficient to drive subsequent tubulointerstitial inflammation (Supplemental Figure 8), and the expression level of VISTA among adoptively transferred T cells was 4% (Supplemental Figure 9). The T cell numbers were not different between WT and *Vsir–/–* mouse–derived T cells after NTN induction (Figure 8, A and B). The CD44 expression levels were also similar between the 2 cell groups (Figure 8, C and D). When competitive proliferation was evaluated in an in vivo model, the cell number and activation marker levels were not different between WT and *Vsir–/–* mouse–derived T cells (Figure 8, E–G). The nonsignificant or minimal effects of VISTA at the end of the T cells might be because its protein expression level was very low in kidney-infiltrated T cells (Figure 1G and Supplemental Figure 9).

### VISTA expression in human glomerulonephritis.

**Figure 9:**
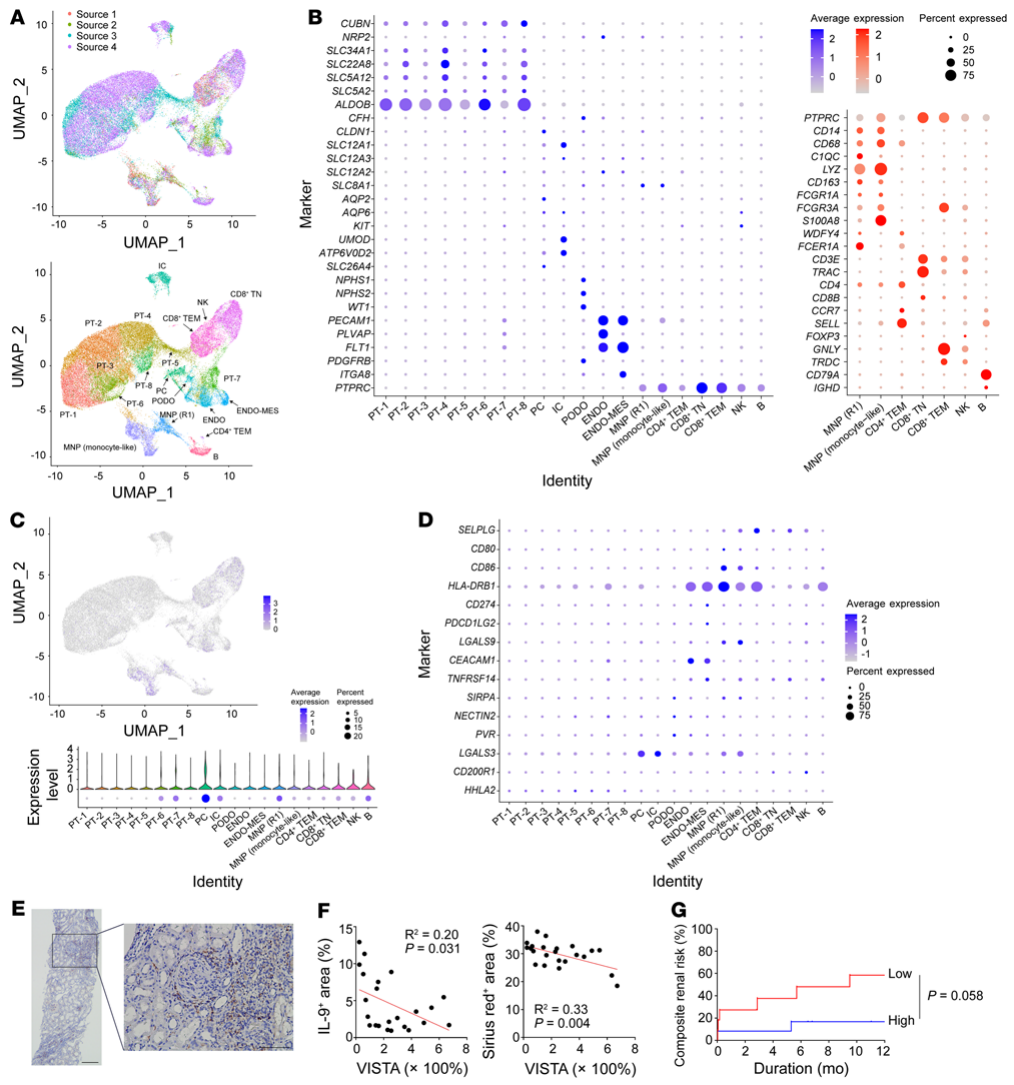
Translation of results to human glomerulonephritis. (**A**) UMAP plots of 55,107 cells from pooled data. (**B**) Dot plots to identify clusters of parenchymal tissue cells (left) and immune cells (right). ENDO, endocyte; MES, mesangial cell. (**C**) Expression of the *C10orf54* gene in the UMAP plot (upper) and clusters (lower). (**D**) Dot plot for the gene expression of PSGL-1 and other immune checkpoints. (**E**) Representative image of kidney sections with ANCA vasculitis immunostained for VISTA. Scale bars: 200 μm (left); 100 μm (right). (**F**) Correlations of VISTA expression with the IL-9^+^ (left) and Sirius red^+^ (right) areas in ANCA vasculitis. The correlation coefficients were measured using Pearson’s correlation test. (**G**) Kaplan-Meier curve of composite renal risk according to VISTA expression. Composite renal risk was defined as a doubling of serum creatinine, development of end-stage renal disease, or death. Statistical significance for survival was calculated using the Kaplan-Meier method with the log-rank test.

It was determined which cell types expressed VISTA within human kidneys using scRNA-sequencing data (29–32). When *PTPRC*^+^ immune cells were examined, chromosome 10 open reading frame 54 (*C10orf54*; human gene name of *Vsir*) was expressed in clusters such as mononuclear phagocytes (MNPs), CD4^+^ T cells, and CD8^+^ T cells (Figure 9, A–C). MNPs were clustered into 2 subsets: one had a gene expression pattern similar to that of mouse R1 macrophages based on *C1QC*, *APOE*, *CD74*, and *HLA-DRA* (named R1-like MNPs), whereas the other had an expression pattern similar to that of mouse monocytes based on *S100A8*, *S100A9*, *FCN1*, *VCAN*, and *IFITM2* (named monocyte-like MNPs; refs. 10, 30 and Supplemental Figure 10). Among them, monocyte-like MNPs had the highest expression, and R1-like MNPs ranked second. Among nonimmune cells, endothelial cells had high expression of *C10orf54*, but protein expression was not evident in human kidneys (Figure 9C and Supplemental Figure 11). In humans, P-selectin glycoprotein ligand-1 (PSGL-1) is known to be a receptor for VISTA in acidic environments (33). When the gene expression of this receptor (named *SELPLG*) was profiled, CD4^+^ and CD8^+^ EM T cell clusters had the highest expression, followed by MNPs and NK cells (Figure 9D). These results suggest that human kidney MNPs interact with other immune cells (particularly EM T cells) via a VISTA/PSGL-1 pair in acidic environments, such as glomerulonephritis (34). The gene expression profiles of other immune checkpoints in normal human kidney cells are shown in Figure 9D.

To translate the mouse results to human disease, we selected human cases with biopsy-proven ANCA vasculitis because both the histological (e.g., crescentic glomerulonephritis and tubulointerstitial fibrosis) and clinical (e.g., rapidly progressive glomerulonephritis) signs are the same as those observed in the NTN model (35–37). VISTA-producing cells were primarily identified in infiltrated immune cells (Figure 9E). VISTA expression was negatively related to the IL-9^+^ and Sirius red^+^ areas (Figure 9F). When 23 samples were divided into 2 groups by the median value of expression (i.e., VISTA^hi^ and VISTA^lo^ kidneys), the VISTA^hi^ group seemed to have a lower risk of renal progression than the VISTA^lo^ group (Figure 9G). These results indicate that VISTA could be a biomarker related to glomerulonephritis and its fibrotic outcome.

## Discussion

Tubulointerstitial fibrosis after glomerular injury determines patient outcomes, but its immunological link and relevant sentinel molecules are not completely understood. The present results may provide clues, and VISTA was identified as a sentinel molecule particularly expressed in kidney macrophages. If VISTA was not expressed in the kidney, immunometabolic changes occurred, such as high oxidative phosphorylation and fatty acid metabolism and IFN-γ overproduction. These changes provoked IL-9 production in surrounding parenchymal tissue cells and aggravated interstitial fibrosis. The protective role of VISTA in tubulointerstitial fibrosis via the IFN-γ/IL-9 axis after glomerular injury could be reflected in human glomerulonephritis, wherein VISTA^hi^ kidneys had low fibrosis and better prognosis than VISTA^lo^ kidneys.

VISTA is an immunoregulatory and inhibitory checkpoint expressed in cells of the myeloid and lymphoid lineages (38). The expression can be inducible in inflammatory or cancerous conditions (12–15), but an interesting aspect is its high expression in kidney-resident macrophages (10). When the expression level was compared among tissue-resident macrophages, the kidney-resident subset had the highest expression, followed by peritoneal, splenic, and bone marrow macrophages (10). These results indicate that the tissue environment may determine VISTA expression in macrophages, and the unique pattern of kidney-resident macrophages implies their homeostatic role under normal conditions or during kidney inflammation. Our previous work identified kidney-resident macrophages as playing a repair role after ischemic tubular damage, and VISTA was determined to be a molecule relevant to this repair role (10). The transition from acute glomerular injury to tubulointerstitial fibrosis was aggravated when VISTA was depleted, wherein immunometabolic changes in T cells were observed, such as augmented oxidative phosphorylation, fatty acid metabolism, and IFN-γ production. These changes may be dependent on tissue status or specific to diseases such as severe glomerulonephritis.

In addition to direct damage by immune cells, the responses of parenchymal tissue cells, including glomeruli, tubules, and other stromal cells, are important for determining kidney outcomes (39). Parenchymal tissue cell–derived cytokines can be inflammatory or antiinflammatory, and all of these cytokines participate in tubulointerstitial fibrosis as a repair or sometimes ongoing process (40, 41). VISTA depletion augmented IFN-γ production from interstitial T cells, and then IL-9 was overwhelmed by surrounding parenchymal tissue cells. IL-9, as a Th2-type cytokine, has been related to immune tolerance and tissue fibrosis (42, 43). IL-9 can be produced in kidneys after injury (44), but the subsequent pathophysiology by IL-9 is less understood than that in other organs, such as the lung and liver. The all-or-none status of IL-9 in transgenic and knockout mice results in unexpectedly reduced and increased inflammatory responses, respectively (45, 46), indicating that proper or specific window levels may be needed to maintain homeostasis of the kidney or repair after injury. However, VISTA-depleted kidneys and subsequent high IFN-γ–producing T cells increased IL-9 levels in surrounding parenchymal tissue cells, which ultimately resulted in overzealous tubulointerstitial fibrosis.

VISTA can tune the activity of T cells in a contact manner, but there is a gap in understanding human kidneys. scRNA profiling indicated that *C10orf54* was transcribed primarily in kidney MNPs and T cells, which was similar to the mouse counterpart. However, gene expression levels were different such that human monocyte-like MNPs (R2 or monocytes in the mouse kidney) had the highest expression levels among immune cell subsets, higher than those of R1-like MNPs (R1 in the mouse kidney). The expression level within kidneys was associated with the fibrotic outcomes of ANCA vasculitis, a severe form of glomerulonephritis. These results will help us understand the difference between mice and humans and could improve intervention strategies related to VISTA or kidney immune cells.

A clear understanding of the pathogenesis of glomerulonephritis will provide valuable insight into disease progression and options for intervention. The present study shows that VISTA, which is primarily expressed in kidney macrophages, has a protective role in tubulointerstitial fibrosis after acute glomerular injury and that IFN-γ and IL-9 are involved in the pathological process. VISTA, as an immunoregulatory sentinel molecule, may be highlighted in future biomarker research and therapy development.

## Methods

### Mice.

C57BL/6 (B6), B6. CD45.1^+^, B6. *Ifng–/–*, and B6. *Rag1–/–* mice were purchased from the Jackson Laboratory (Jax). B6. *Vsir–/–* mice were established and bred using a cryopreserved sperm (KOMP Repository, UCD, Davis, California, USA). CD45.1^+^ *Vsir–/–* mice were generated to identify the cell origin in adoptive transfer experiments. All mice used in the experiments were 8 weeks of age and male. Animals were housed under specific pathogen–free conditions at the facility of Seoul National University College of Medicine.

For the NTN model, mice were preimmunized with 200 μg of sheep immunoglobulin G in Freund’s complete adjuvant via the intraperitoneal route, followed by 5 μL/g sheep nephrotoxic serum (Probetex Inc.) via intravenous injection 4 days later. To evaluate the IFN-γ/IL-9 axis, recombinant proteins (5 ng of IFN-γ or IL-9) were treated via subcapsular injection at day 7 after NTN induction, and antibodies (1 mg of anti–IFN-γ or anti–IL-9 antibody) were administered via intravenous injection at day 0 of NTN induction. As a metabolic inhibitor, 10 ng of oligomycin (Sigma Aldrich) or etomoxir (Sigma Aldrich) was administered via subcapsular injection at day 7 after NTN induction. The mice were sacrificed at the indicated time points after reperfusion.

### Flow cytometry.

Cells were washed, resuspended in staining buffer consisting of 2% horse serum and 0.05% sodium azide, blocked with anti-mouse CD16/CD32 antibodies for 15 minutes, and then incubated with primary antibodies. Alternatively, following surface staining, cells were incubated with fixation-permeabilization buffer, washed with permeabilization buffer (Fixation/Permeabilization Solution Kit; BD Biosciences), and then incubated with antibodies against intracellular antigens. Samples were processed by a BD Fortessa (BD Biosciences) and analyzed with FlowJo software. The antibodies used for flow cytometry are listed in Supplemental Table 1.

### Immunofluorescence.

Paraffin-embedded tissue sections were deparaffinized and rehydrated with xylene and ethanol. After retrieval of antigens, sections were blocked in 0.1 M Tris containing 0.1% Triton X-100, 10% normal mouse serum, and 1% bovine serum albumin. Images were acquired using a Leica DMi8 microscope (Leica Microsystems). The contact analysis between macrophages and infiltrated T cells was performed using the Imaris program (version 9.3.1; Bitplane). The colocalization module (*Coloc* in Imaris) was used to label CD3 and DAPI for T cells and F4/80 and CD72 for kidney-resident macrophages. Surfaces and spots were created for T cells and macrophages, and the number of spots was calculated within the indicated distance from the surface. The antibodies used for immunofluorescence are listed in Supplemental Table 1.

### Immunohistochemistry.

The steps before blocking were the same as those described in the immunofluorescence section. Blocking reagent was added for 1 hour at room temperature. Images were obtained using a Leica TCS SP8 STED CW (Leica Microsystems). All morphometric parameters were determined using a microscope coupled to a computerized morphometry system (Qwin3, Leica). The antibodies used for immunofluorescence are listed in Supplemental Table 1.

### Real-time qPCR.

RNA was purified from tissues using a Direct-zol RNA MicroPrep Kit (Zymo Research). RNA was reverse transcribed to cDNA with a PrimeScript RT Reagent Kit (TaKaRa Bio Inc.). Gene expression was evaluated by real-time reverse-transcriptase PCR using iQ SYBR Green Supermix (Bio-Rad) on a PCR amplification and detection instrument (CFX Connect Real-Time PCR Detection System; Bio-Rad). Gene expression was normalized to *18S* *rRNA*, and the mean relative gene expression was calculated using the 2^–ΔΔCt^ method. The primers used are listed in Supplemental Table 2.

### Single-cell RNA sequencing.

After digesting kidneys with 0.2 mg/ml liberase (Roche) at 37°C for 30 minutes, CD45^+^ cells were flow cytometrically sorted for the subsequent process. Libraries were prepared using the chromium controller according to the 10× Chromium Next GEM Single Cell 3′ protocol (version 3.1; 10× Genomics). Briefly, the cell suspensions were diluted in nuclease-free water to achieve a target count of 10,000. The cell suspension was mixed with master mix and loaded with gel beads and partitioning oil into a Chromium Next GEM Chip G. RNA transcripts were uniquely barcoded and reverse-transcribed within droplets. The cDNA molecules were pooled and subjected to an end-repair process, the addition of a single A base, and ligation of the adapters. The products were purified and enriched using PCR to produce the final cDNA library. The purified libraries were quantified using quantitative PCR (qPCR) according to the manufacturer’s protocol (Sigma-Aldrich) and were qualified using the Agilent 4200 TapeStation (Agilent Technologies) and sequenced using HiSeqX (Illumina) with a read length of 28 bp for read 1 (cell barcode and UMI), an 8 bp index read (sample barcode), and a read length of 91 bp for read 2 (RNA read). The Cell Ranger pipeline (version 3.1.0; 10× Genomics) was used to process the raw data set.

As a quality control of pooled scRNA-sequencing data of normal mouse kidneys (30, 47, 48), genes expressed in 10 or more cells, cells with 200–4000 unique genes, and cells with 20% or more mitochondrial genes were used. In the flow cytometrically sorted immune cells for comparison between NTN-induced WT and *Vsir–/–* mice, genes expressed in 5 or more cells, cells with 200 or more UMI counts, and cells with 10% or fewer mitochondrial genes were selected. As a quality control of pooled scRNA-sequencing data of normal human kidneys (29–32), the same quality control as in the NTN model was used. The raw data on normal mouse and human kidneys could be identified in the original papers, and the data on NTN-induced kidneys were deposited in the NCBI’s Gene Expression Omnibus database (GEO GSE181163).

R (version 4.0.3; The Comprehensive R Archive Network: http://cran.r-project.org) and Seurat (version 3.2.3) packages were used in analyses, as follows: SCTransform, merging data with adjusting batch-effects (49); FindNeighbors and FindClusters, finding clusters; RunUMAP, FeaturePlot, DotPlot, and VlnPlot, visualizing data; Findmarkers, identifying differentially expressed genes; fgsea, analyzing the Hallmark gene set enrichment analysis; and SingleCellSignalR, evaluating the ligand-receptor interaction (50).

### Kidney histology.

Kidneys were fixed in 4% formalin, paraffin embedded, and cut into 4 μm sections. Sections were then stained with periodic acid–Schiff before microscopy analysis (Leica Microsystems). Glomerular injury was estimated in a blinded manner based on the following system: 0, normal glomeruli without structural damage; 1, glomerular matrix expansion and edema formation of less than 25% of the glomeruli; 2, increased intraglomerular cell count and swelling up to 50%; 3, obliteration or collapse in up to 75% of glomeruli; and 4, complete structural obliteration and thrombosis (51). The assessment of tubular injury in the cortex was graded in a blinded manner based on the percentages of tubular dilatation, cast deposition, brush border loss, and necrosis as follows: 0, no involvement; 1, less than 10% involvement; 2, 10%–25% involvement; 3, 25%–50% involvement; 4, 50%–75% involvement; and 5, more than 75% involvement. Ten random fields per kidney section (×200 magnification) were used for quantification. Sirius red (Abcam) and Masson’s trichrome (Roche) staining assays were performed according to the manufacturers’ recommendations. Apoptotic cells were identified in tissue sections using the Click-iT TUNEL imaging assay (Thermo Fisher Scientific) according to the manufacturer’s instructions. The areas of fibrosis and positive staining were evaluated using ImageJ software (version 1.5; NIH).

### Adoptive transfer of T cells.

CD62L^+^CD4^+^ or CD62L^+^CD8^+^ T cells were negatively sorted from lymph nodes using anti-biotin microbeads (Miltenyi Biotec). The purity was greater than 95%. A total of 5 × 10^6^ cells from WT or *Vsir–/–* mice (or *Ifng–/–* mice in other experiments) were adoptively transferred to *Rag1–/–* mice at day 2 after NTN induction. For a competitive assay, 2.5 × 10^6^ and 2.5 × 10^6^ cells from CD45.2^+^ WT and CD45.1^+^ *Vsir–/–* mice, respectively, were adoptively transferred to *Rag1–/–* mice under the same scheme.

### Human samples.

Kidney biopsied tissues were evaluated in patients with ANCA vasculitis (*n* = 23). Immunohistochemistry and Sirius red staining were used to estimate the VISTA^+^, IL-9^+^, and fibrotic areas in the interstitium of the cortex. Composite renal risk (e.g., doubling of serum creatinine, development of end-stage renal disease, and death) was compared between the VISTA^hi^ and VISTA^lo^ groups. Normal human kidney tissue was obtained from patients who underwent radical nephrectomy due to urogenital malignancy, but did not have hydronephrosis or any infectious diseases.

### Statistics.

All analyses and calculations were performed using GraphPad Prism (version 8.0; GraphPad Software). The results are expressed as the mean ± SEM or proportion. Differences between groups were evaluated using 2-tailed Student’s *t* test and ANOVA with Tukey’s test for comparison between 2 and multiple groups, respectively. Survival curves were drawn using the Kaplan-Meier method. To compare survival curves between groups, the log‑rank test was applied. A *P* value of less than 0.05 was considered statistically significant.

### Study approval.

All animal experiments were approved by the Seoul National University IACUC (no. SNU-190621-3-13). The study design for human samples was approved by the IRB of Seoul National University Hospital (H-1810-016-975) and complied with the Declaration of Helsinki. All patients provided written, informed consent for the donation and use of their specimens.

## Author contributions

SSH and DSL designed the study. MGK, CLK, MH, and JH performed experiments and analyzed data. DY and SSH analyzed bioinformatics data. KCM and SSH supervised histology studies. CWJ, CK, and DKK collected data. DKK, KHO, KWJ, and YSK provided critical reading of the manuscript. MGK, DSL, and SSH wrote the manuscript. All authors approved the final manuscript. Co–first authorship order was determined based on contribution to results generation.

## Supplementary Material

Supplemental data

Supplemental data set 1

## Version 1 11/09/2021

In-Press Preview

## Version 2 01/04/2022

Electronic publication
