# Supplementary material for: Kidney VISTA prevents IFN-γ/IL-9 axis–mediated tubulointerstitial fibrosis after acute glomerular injury
Source: J Clin Invest. 2022 Jan 4;132(1):e151189. doi: 10.1172/JCI151189 (PMC8718152; doi:10.1172/JCI151189)

Kidney VISTA prevents IFNγ-IL-9 axis-mediated tubulointerstitial fibrosis after acute glomerular injury

Min-Gang Kim, Donghwan Yun, Chae Lin Kang, Minki Hong, Ju Hyun Hwang, Kyung Chul Moon, Chang Wook Jeong, Cheol Kwak, Dong Ki Kim, Kook-Hwan Oh, Kwon Wook Joo, Yon Su Kim, Dong-Sup Lee and Seung Seok Han

Supplementary Tables 1–2

Supplementary Figures 1–11

Supplementary Table 1. Antibodies and materials used in this study

Antibodies	Source	Clone
Anti-mouse/human CD11b antibody-BV785	BioLegend	M1/70
Anti-mouse F4/80 antibody-BV711	BioLegend	T45-2342
Anti-mouse CD3 antibody-BV711	BioLegend	17A2
Anti-mouse CD8 antibody-BV650	BioLegend	53-6.7
Anti-mouse CD62L antibody-BV605	BioLegend	MEL-14
Anti-mouse I-A/E antibody-BV605	BioLegend	M5/114.15.2
Anti-mouse CD44 antibody-AmCyan	BioLegend	1M7
Anti-mouse CD4 antibody-AmCyan	BioLegend	RM4-5
Anti-mouse CD45.2 antibody-PerCP-Cy5.5	BioLegend	104
Anti-mouse B220 antibody-FITC	BD Biosciences	RA3-6B2
Anti-mouse CD72 antibody-FITC	BD Biosciences	K10.6
Anti-mouse IL-4 antibody-PE	BD Biosciences	11B11
Anti-mouse IL-17 antibody-PE	eBioscience	17B7
Anti-mouse CD19 antibody-PE-Cy7	BioLegend	6D5
Anti-mouse CD4 antibody-PE-Cy7	BioLegend	GK1.5
Anti-mouse Ly6C antibody-PE-Cy7	BioLegend	HK1.4
Anti-mouse CD11b antibody-PE-Cy7	eBioscience	M1/70
Anti-mouse IFNγ antibody-APC	BioLegend	XMG1.2
Anti-mouse IL-9 antibody-APC	BioLegend	RM9A4
Anti-mouse VISTA antibody-APC	BioLegend	MH5A
Anti-mouse NK1.1 antibody-APC	BioLegend	PK136
Anti-mouse Ly6G antibody-APC-Cy7	BioLegend	1A8
Anti-mouse CD8 antibody-APC-Cy7	BioLegend	53-6-7
Anti-mouse CD45.1 antibody-APC-Cy7	BioLegend	A20
Anti-mouse CD4 antibody-APC-Cy7	BioLegend	GK1.5
Anti-mouse NK1.1 antibody-APC	BioLegend	PK136
Anti-mouse F4/80 antibody-APC	BioLegend	BM8
Anti-mouse/human VISTA antibody-AF488	Pharmabcine	2C12
Human IgG1 isotype control antibody-AF488	Pharmabcine	2G4

Anti-mouse CD16/CD32 antibody	eBioscience	93
Anti-human Fc receptor binding inhibitor	eBioscience	Polyclonal
Fixable Viability Stain 450	BD Biosciences	
Fixable Viability Stain 780	BD Biosciences	
Anti-mouse IFNγ antibody	BioXcell	XMG1.2
Anti-mouse IL-9 antibody	BioXcell	9C1
Anti-mouse Thy1 antibody	Ours	T24
Anti-mouse IL-9 antibody	Abcam	Polyclonal
Anti-mouse IL-9 receptor antibody	Thermo Fisher Scientific	Polyclonal
Anti-mouse αSMA antibody	Thermo Fisher Scientific	17HCLC
Anti-mouse VISTA antibody	Cell Signaling	D5L5T
Anti-mouse VISTA antibody	BioXcell	13F3
Anti-mouse F4/80 antibody	Abcam	BM8
Anti-mouse CD3 antibody	Abcam	SP162
Anti-mouse CD72 antibody	R&D Systems	Polyclonal
Anti-mouse KIM-1 antibody	Abcam	Polyclonal
Anti-human IL-9 antibody	Abcam	Polyclonal
Anti-human VISTA antibody	Abcam	SP345
Anti-human CD14 antibody	Novus Biologicals	Polyclonal
Anti-human CD3 antibody	Abcam	PS1
Anti-rabbit IgG antibody-HRP	Abcam	
Anti-mouse CD11b antibody-biotin	BD Biosciences	M1/70
Anti-mouse CD45R/B220 antibody-biotin	BD Biosciences	RA3-6B2
Anti-mouse NK1.1 antibody-biotin	BD Biosciences	PK136
Anti-mouse CD8a antibody-biotin	BD Biosciences	53-6.7
Anti-mouse CD4 antibody-biotin	BD Biosciences	RM4-5
Rabbit IgG isotype control antibody	Thermo Fisher Scientific	
Anti-rat IgG cross-adsorbed secondary antibody-AF 594	Thermo Fisher Scientific	Polyclonal
Anti-rat IgG cross-adsorbed secondary antibody-AF 488	Thermo Fisher Scientific	Polyclonal
Anti-goat IgG cross-adsorbed secondary antibody-AF 568	Thermo Fisher Scientific	Polyclonal
Anti-rabbit IgG cross-adsorbed secondary antibody-AF 488	Thermo Fisher Scientific	Polyclonal

Supplementary Table 2. Primers used for real-time qPCR

Genes	Forward (5′–3′)	Reverse (5′–3′)
Ifng	ACAGCAAGGCGAAAAAGGAT	TGAGCTCATTGAATGCTTGG
Gzma	AGACCGTATATGGCTCTACT	CCCTCACGTGTATATTCATC
Tnf	CCTCACACTCACAAACCACCA	GTGAGGAGCACGTAGTCGG
Il1b	ATGCCACCTTTTGACAGTGATG	AGCTTCTCCACAGCCACAAT
Il2	GCAGGATGGAGAATTACAGGA	TCAGAGCCCTTTAGTTTTAC
Il4	TGAACGAGGTCACAGGAGAA	CGAGCTCACTCTCTGTGGTG
Il5	AGCAATGAGACGATGAGGC	ACACTTCTCTTTTTGGCGGT
Il6	TGATGCACTTGCAGAAAACA	ACCAGAGGAAATTTTCAATAGGC
Il8	GATTCACCTCAAGAACATCCAGA	GGACACCTTTTAGCATCTTTTGG
Il9	ATGTTGGTGACATACATCCTTGC	TGACGGTGGATCATCCTTCAG
Il10	ATCGATTTCTCCCCTGTGAA	TGTCAAATTCATTCATGGCCT
Il12b	GATTCAGACTCCAGGGGACA	GGAGACACCAGCAAAACGAT
Il13	ACATCACACAAGACCAGACTCC	GAGGCCATGCAATATCCTCT
Il17a	TCTCCACCGCAATGAAGACC	CACACCCACCAGCATCTTCT
Il22	TTGAGGTGTCCAACTTCCAGCA	AGCCGGACGTCTGTGTTGTTA
Il33	TGGCCTCACCATAAGAAAGG	GACTTGCAGGACAGGGAGAC
Tgfb1	TGACGTCACTGGAGTTGTACGG	GGTTCATGTCATGGATGGTGC
Atp5o	TGACCACAGCATCTCCTCTA	GTCAGTCTTGATCTCCAGTTTCA
Atp6voc	CCCTAGAGTGCTCCTGTGTATAA	GCTCCACAGACGCATGAATAG
Hsp90aa1	CATCGGACGCTCTGGATAAA	CTGTTTGCTGGGAATGAGATTG
Odc1	CTTGTGAGGAGCTGGTGATAAT	GCAGTCAAACTCGTCCTTAGT
18S rRNA	CGGCTACCACATCCAAGGAA	GCTGGAATTACCGCGGCT

Supplementary Figure 1. Baseline comparison between WT and *Vsir*$^{-/-}$ mice (n = 3 per group). (A) Kidney damage markers such as blood urea nitrogen (BUN), serum creatinine (Cr), and random urine protein-to-creatinine ratio (uPCR). (B) Representative PAS staining images of kidneys in WT (upper image) and *Vsir*$^{-/-}$ (lower image) mice. Scale bar = 100 μm. Glomerular and tubular injury scores. (C) Representative Sirius red images of kidneys in WT (upper image) and *Vsir*$^{-/-}$ (lower image) mice. Scale bar = 100 μm. Comparison of the Sirius red^{+} area between WT and *Vsir*$^{-/-}$ kidneys.

(A)

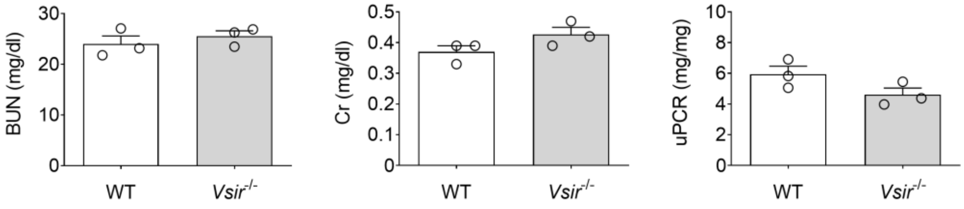

(B)

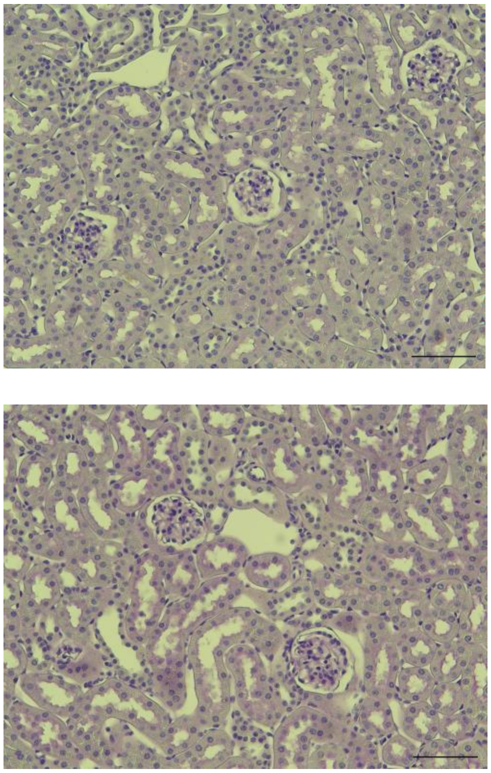

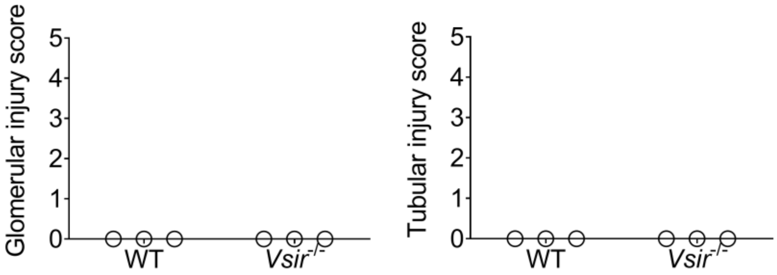
Glomerular injury score
5
4
3
2
1
0
WT
Vsir-/-
Tubular injury score
5
4
3
2
1
0
WT
Vsir-/-

(C)

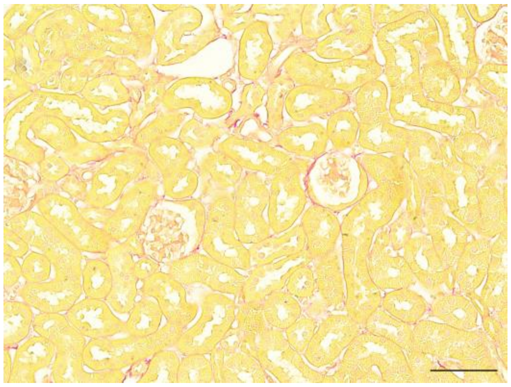

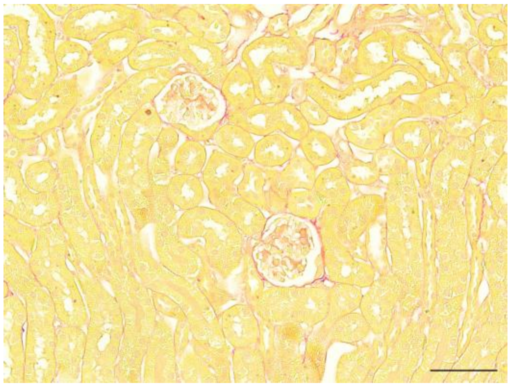

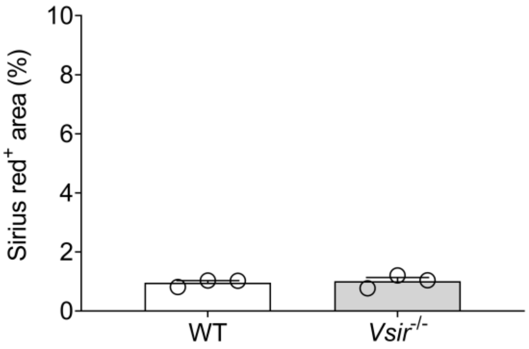
Sirius red+ area (%)
10
8
6
4
2
0
WT
Vsir-/-

Supplementary Figure 2. Serum albumin and cholesterol levels in WT and *Vsir*$^{-/-}$ mice (n = 8 per group). Hypoalbuminemia and hypercholesterolemia are the features of aggravated glomerulonephritis (i.e., signs of nephrotic syndrome). ***P < 0.001. Data were obtained from three independent experiments.

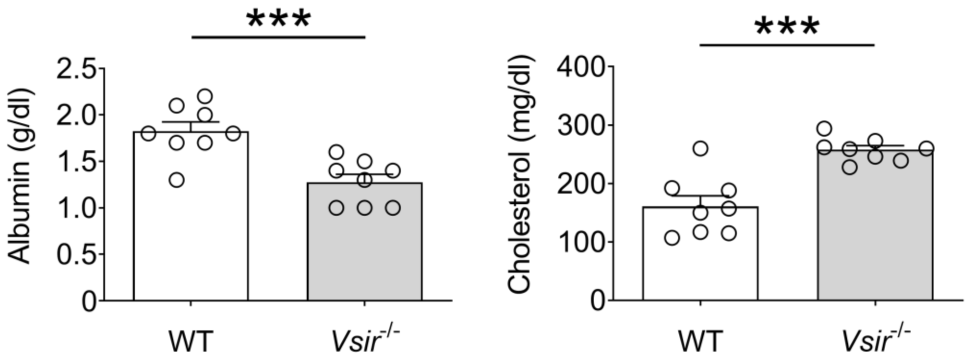

Supplementary Figure 3. Metabolic gene expression in infiltrated T cells. (A) Representative genes related with oxidative phosphorylation. (B) Representative genes related with fatty acid metabolism. Infiltrated T cells were isolated using magnetic bead sorting method ($n = 3$ per group). P values are calculated using the unpaired Student's t test. $^{*}P < 0.05$, $^{**}P < 0.01$.

(A)

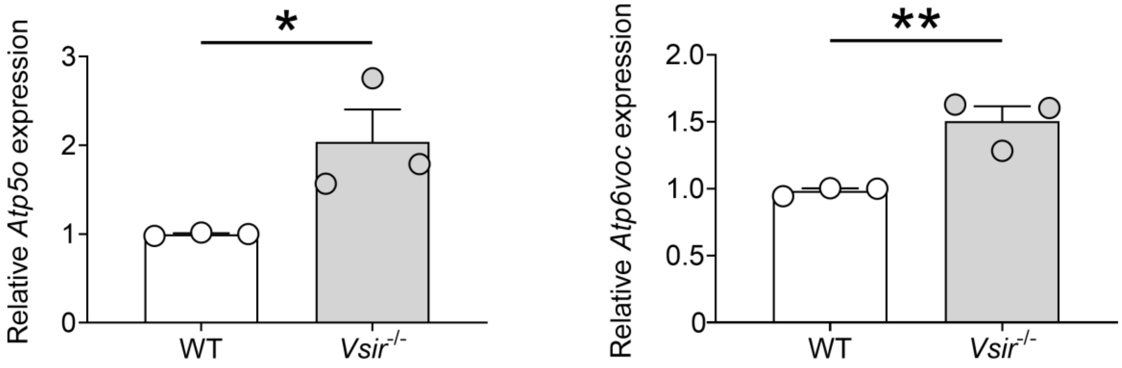

(B)

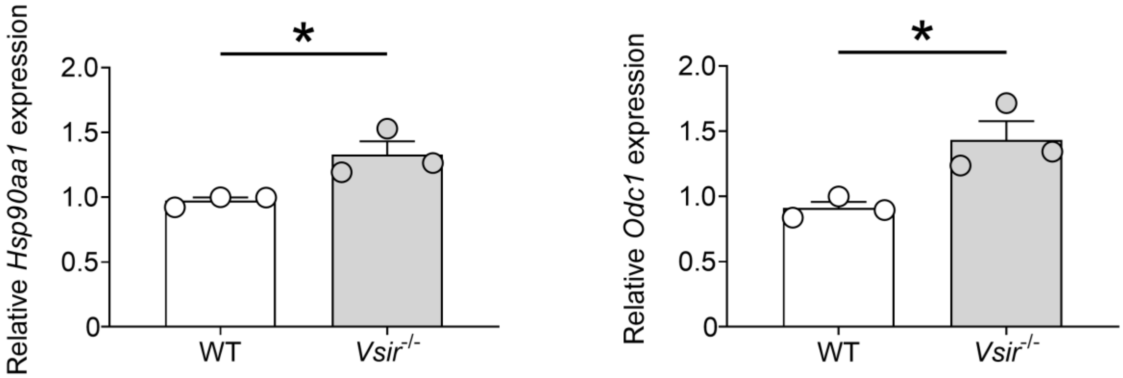

Supplementary Figure 4. Proportions of IFNγ$^+$ cells in CD4$^+$ and CD8$^+$ cells after the use of anti-VISTA antibody (*n* = 4 per group). Either 300 μg of anti-VISTA antibody or control antibody was treated via intraperitoneal injection every 3 days from day 0 of NTN induction. *P* values are calculated using the unpaired Student's *t* test. **P* < 0.05. Data represent two independent experiments.

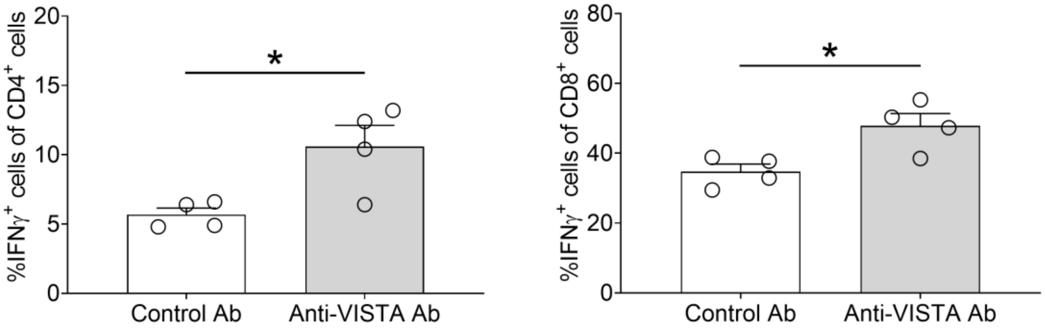

Supplementary Figure 5. Proportions of IFNγ$^+$ cells in CD4$^+$ and CD8$^+$ cells after the treatment with the inhibitors of oxidative phosphorylation and fatty acid metabolism (*n* = 4 per group). 10 ng of oligomycin (OM) and etomoxir (Eto) were treated via subcapsular injection at day 7 after NTN induction to inhibit the oxidative phosphorylation and fatty acid metabolism, respectively. *P* values are calculated using analysis of variance with Tukey's test. **P* < 0.05, ***P* < 0.01. Data represent two independent experiments.

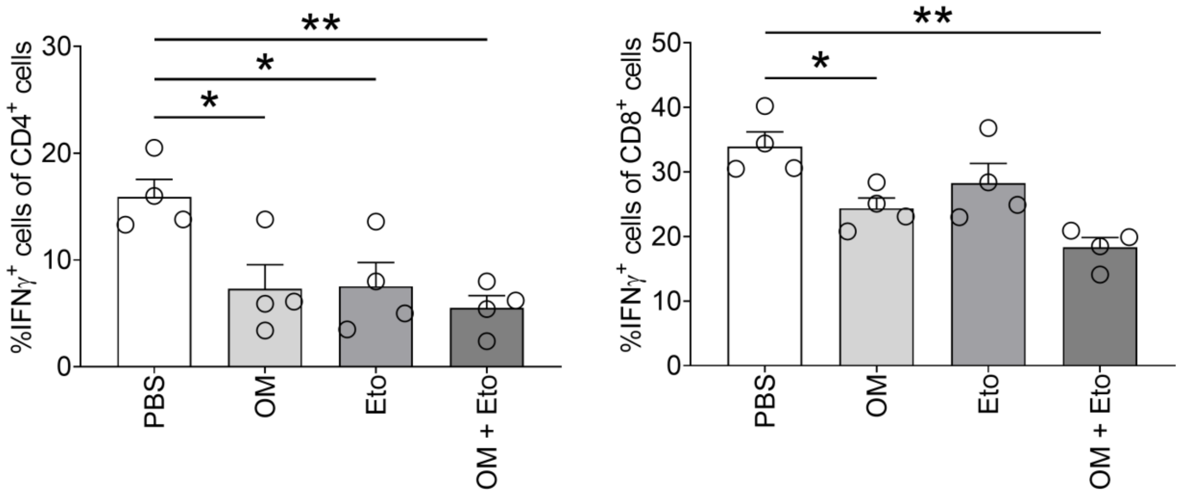

Supplementary Figure 6. T cell-dependency of the NTN model in *Vsir*$^{-/-}$ mice. (A) Flow cytometry confirmation of T-cell depletion after anti-Thy1 antibody. (B) Kidney damage markers after the anti-Thy1 antibody. The isotype control or anti-Thy1 antibody was used before NTN induction. *P < 0.05, **P < 0.01.

(A)

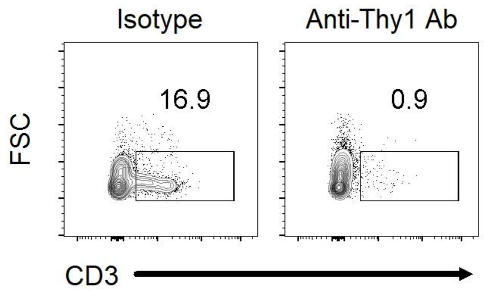

(B)

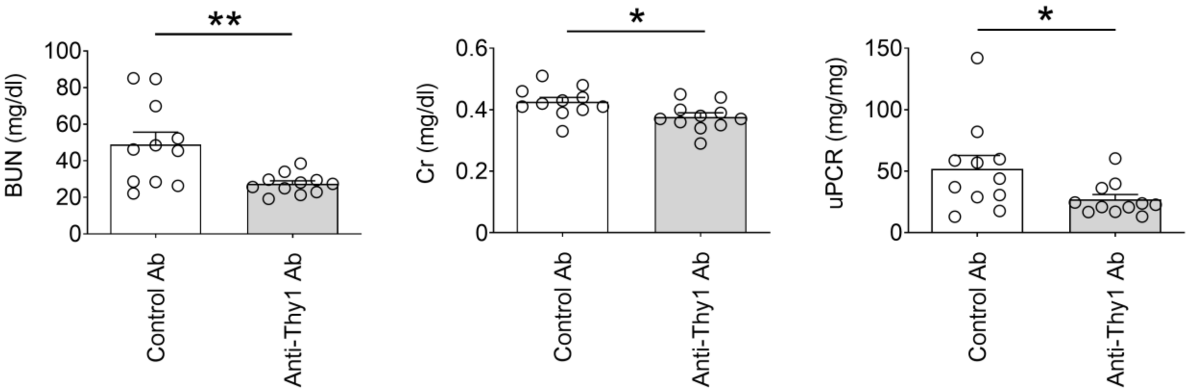

Supplementary Figure 7. Expression of the IL-9 receptor (IL-9R) in NTN-induced kidneys. (A) Representative images of kidney sections immunostained for the IL-9R in WT (upper) and *Vsir*$^{-/-}$ (lower) kidneys. (B) Comparison of the IL-9R^{+} area in NTN-induced WT and *Vsir*$^{-/-}$ kidneys.

(A)

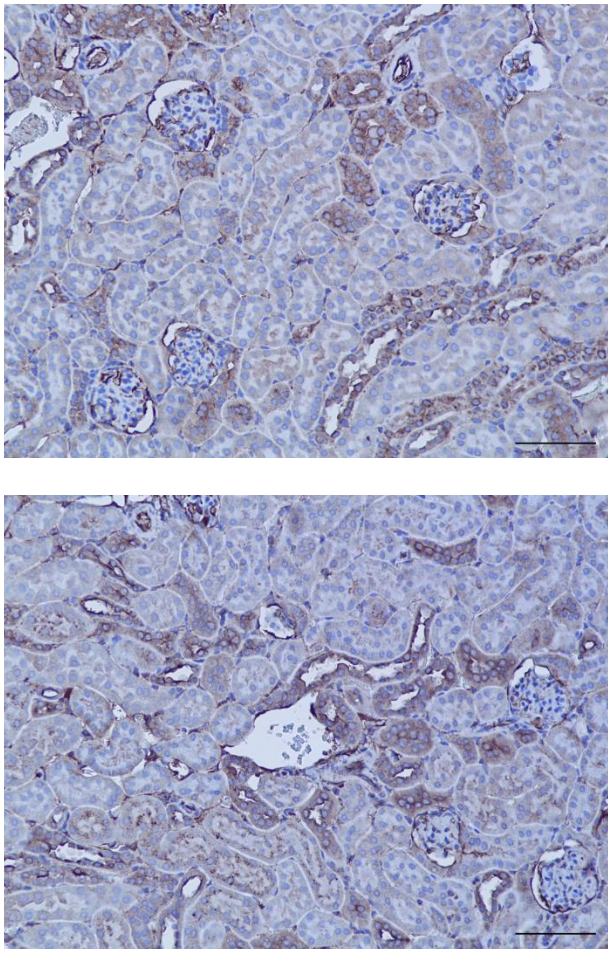

(B)

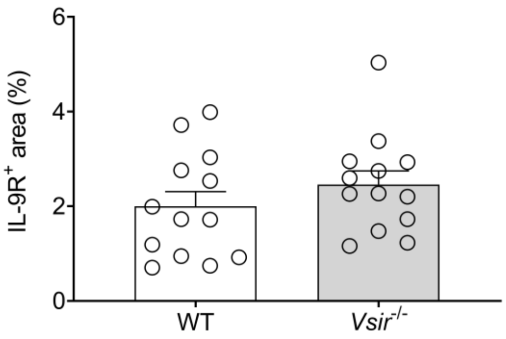

Supplementary Figure 8. Figure. Kidney damage markers in NTN-induced *Rag1*$^{-/-}$ mice with adoptive transfer of T cells. Phosphate buffered saline (PBS) was used as a vehicle. **$P < 0.01$. Data represent two independent experiments.

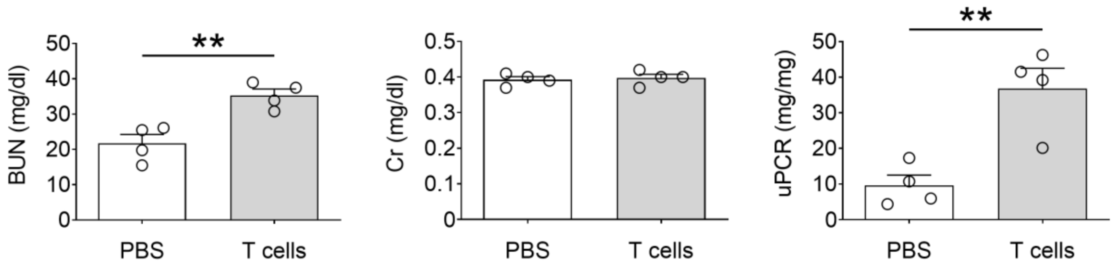

Supplementary Figure 9. Expression of VISTA protein in adoptively transferred T cells from WT mice to NTN-induced *Rag1*$^{-/-}$ mice. A representative flow cytometry plot of VISTA at day 8 after the NTN induction is displayed as obtained from two independent experiments.

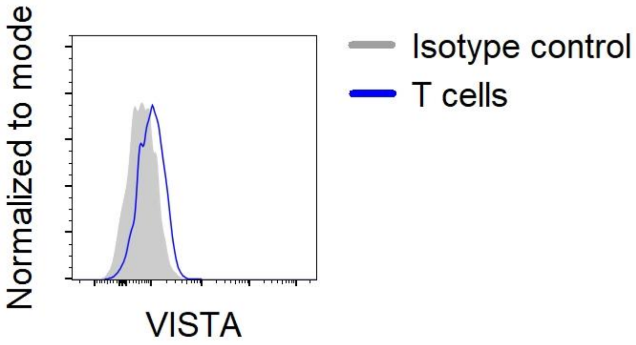

Supplementary Figure 10. Volcano plot showing gene expression changes in two mononuclear phagocyte (MNP) clusters from human kidneys.

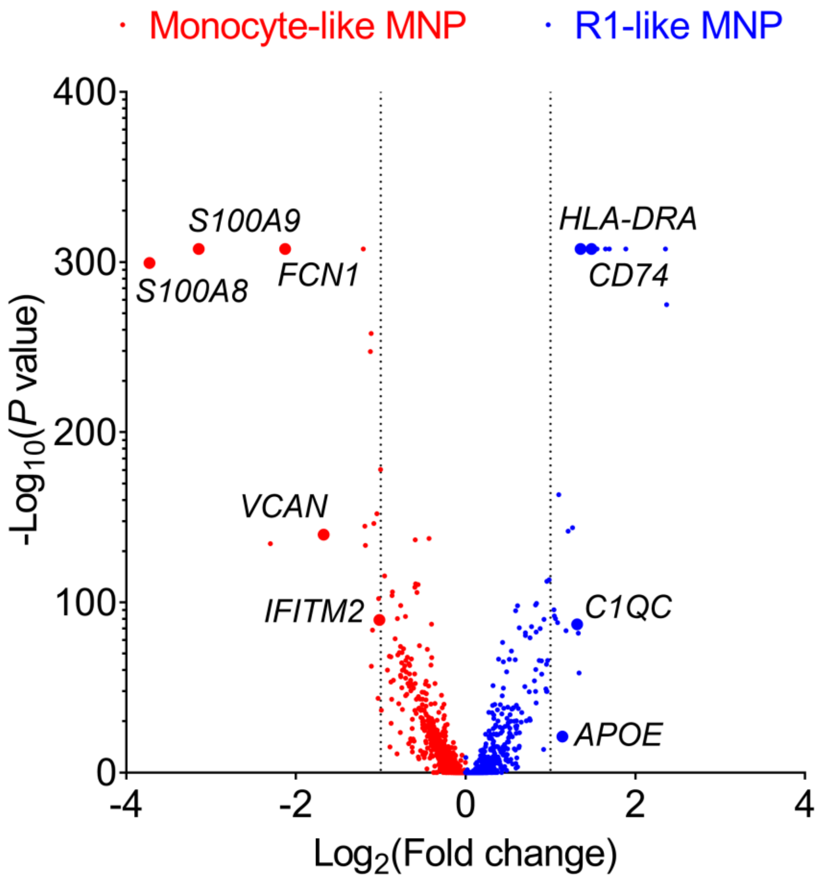

Supplementary Figure 11. Representative image of normal human kidney sections immunostained for VISTA. Scale bar = 200 μm. The image was selected from two independent samples.

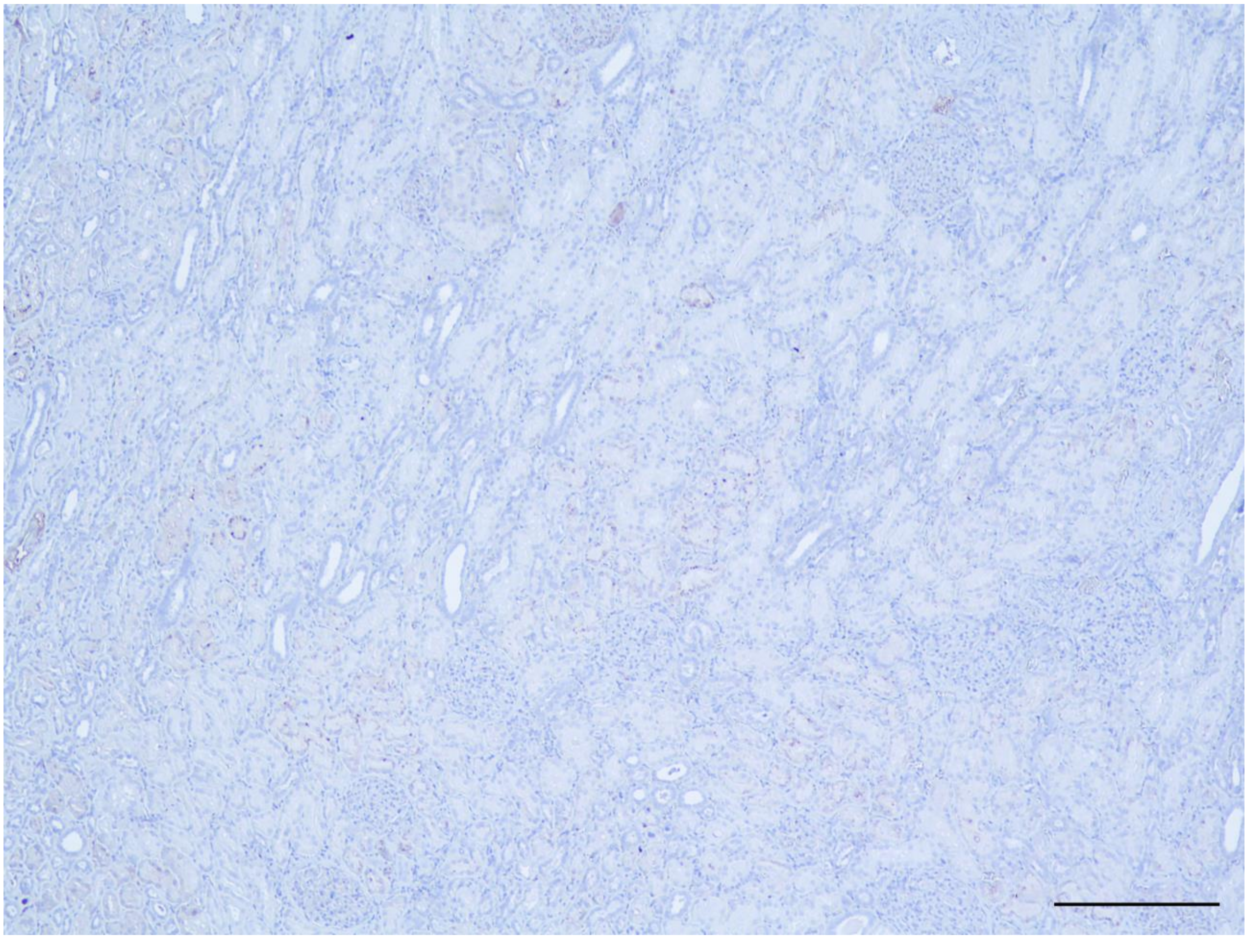

Supplement: Supplemental data [file jci-132-151189-s021.pdf]
